# Supplementary figures and images for: Elicitation with Bacillus QV15 reveals a pivotal role of F3H on flavonoid metabolism improving adaptation to biotic stress in blackberry
Source: PLoS One. 2020 May 6;15(5):e0232626. doi: 10.1371/journal.pone.0232626 (PMC7202615; doi:10.1371/journal.pone.0232626)

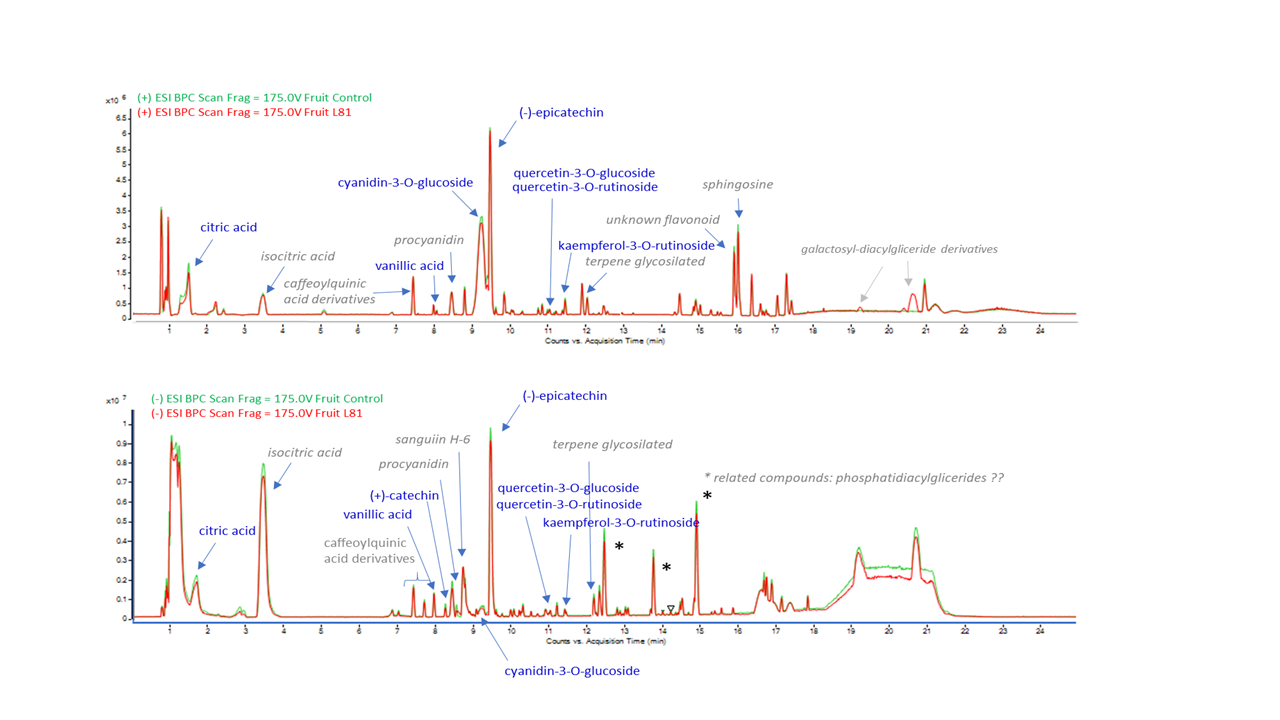

Supplement: S1 Fig — Overlaid Chromatograms (positive and negative ion mode) obtained from LC/MS/TOFF analysis of the methanolic extract of BlackBerry fruit samples. Control samples are represented in green while QV15 samples appear in red. (TIF) [file pone.0232626.s001.tif]
