# Supplementary material for: Elicitation with Bacillus QV15 reveals a pivotal role of F3H on flavonoid metabolism improving adaptation to biotic stress in blackberry
Source: PLoS One. 2020 May 6;15(5):e0232626. doi: 10.1371/journal.pone.0232626 (PMC7202615; doi:10.1371/journal.pone.0232626)
Supplement: S1 Table — (DOCX) [file pone.0232626.s002.docx]

**Supplementary material**

**Table S1.** Number of mappable samples and paired readings per sample. (Supplementary material).

| Sample Name | Total reads | Mapped reads | %Mapped reads | HQ reads | %Hq reads | Properly paired reads | %Properly paired reads | Splice reads | %Splice reads |
| --- | --- | --- | --- | --- | --- | --- | --- | --- | --- |
| Control Leaves 1 | 86368754 | 43670859 | 50.56 | 22284674 | 25.8 | 22284674 | 25.8 | 7294564 | 8.45 |
| Control Leaves 2 | 87959902 | 43952114 | 49.97 | 22093122 | 25.12 | 22093122 | 25.12 | 7139829 | 8.12 |
| Control Leaves 3 | 99213134 | 50802855 | 51.21 | 26619938 | 26.83 | 26619938 | 26.83 | 8187638 | 8.25 |
| Control Fruit 1 | 130528148 | 66264548 | 50.77 | 34046268 | 26.08 | 34046268 | 26.08 | 11867506 | 9.09 |
| Control Fruit 2 | 1046011910 | 53621202 | 51.26 | 26740362 | 25.56 | 26740362 | 25.56 | 9291592 | 8.88 |
| Control Fruit 3 | 84392948 | 42792000 | 50.71 | 21516114 | 25.5 | 21516114 | 25.5 | 7514226 | 8.9 |
| QV15 Leaves 1 | 185999008 | 96998075 | 52.15 | 50825372 | 27.33 | 50825372 | 27.33 | 15367448 | 8.26 |
| QV15 Leaves 2 | 170265810 | 89277463 | 52.43 | 46949912 | 27.57 | 46949912 | 27.57 | 14620244 | 8.59 |
| QV15 Leaves 3 | 106205034 | 55335242 | 52.1 | 29675430 | 27.94 | 29675430 | 27.94 | 9499377 | 8.94 |
| QV15 Fruit 1 | 88992442 | 45637233 | 51.28 | 23777998 | 26.72 | 23777998 | 26.72 | 8124065 | 9.13 |
| QV15 Fruit 2 | 98240804 | 50753378 | 51.66 | 27006822 | 27.49 | 27006822 | 27.49 | 9153643 | 9.32 |
| QV15 Fruit 3 | 132291254 | 67018800 | 50.66 | 34152236 | 25.82 | 34152236 | 25.82 | 11960774 | 9.04 |
