# Supplementary material for: Elicitation with Bacillus QV15 reveals a pivotal role of F3H on flavonoid metabolism improving adaptation to biotic stress in blackberry
Source: PLoS One. 2020 May 6;15(5):e0232626. doi: 10.1371/journal.pone.0232626 (PMC7202615; doi:10.1371/journal.pone.0232626)
Supplement: S2 Table — Glutathione S transferases gene expression analyzed by RT-qPCR in leaves and fruit. Asterisks indicate significant differences, according to Fisher test (p<0.05). (DOCX) [file pone.0232626.s003.docx]

**Supplementary material**

**Table S2**. **Glutathione S transferases** gene expression analyzed by RT-qPCR in leaves and fruit. Asterisks indicate significant differences, according to Fisher test (p<0.05).

|  | **Glutathione S transferases**  **Leaves differential gene expression** | **Glutathione S transferases**  **Fruit differential gene expression** |
| --- | --- | --- |
| **GST1** | 0.06 ± 0.045* | 1.23 ± 0.082* |
| **GST2** | 1.81 ± 0.382* | 0.01 ± 0.004* |
