# Supplementary material for: Elicitation with Bacillus QV15 reveals a pivotal role of F3H on flavonoid metabolism improving adaptation to biotic stress in blackberry
Source: PLoS One. 2020 May 6;15(5):e0232626. doi: 10.1371/journal.pone.0232626 (PMC7202615; doi:10.1371/journal.pone.0232626)
Supplement: S3 Table — (DOCX) [file pone.0232626.s004.docx]

**Supplementary material**

**Table S3.** Primers designed to RT-qPCR expression analysis (Barriuso et al., 2008a; Dos Santos et al., 2003; Efrose et al., 2012; García-Seco et al., 2015; Janda et al., 2015).

| Gene | Forward primer | Reverse primer |
| --- | --- | --- |
| *Ru4CL* | 5´CCAGAAGTTCGAGATCAACAAGT | 5´GTCGGGACACTTGGTAATAGACA |
| *RuACT* | 5´ATGTTCCCTGGTATTGCAGAC | 5´CCACAACCTTGATCTTCATGC |
| *RuANR* | 5´TCGCAATGTACTTCCAAGAAAC | 5´CTTCATCAGCTTACGGAAATCAC |
| *RuANS* | 5´TTGGTCTGGGATTAGAAGAAAGG | 5´CTGAGGGCATTTTGGGTAGTAAT |
| *RuC4H* | 5´CATCTGTAGGGAAGTGAAGGAGA | 5´ACTTCAACCCTTCGTTAGTTGTG |
| *RuCHI1* | 5´CAAGAAGGATTCCATCATCACA | 5´CTCCACTTTGATCTTTGACGACT |
| *RuCHI2* | 5´GAGGCAGTTCTTGAGTCAATCAT | 5´CACGCTATCATCACTCACTTTCA |
| *RuCHS* | 5´ATGGTGGTTGTTGAAATTCC | 5´CTGGATTGCACACCCAGGTGGCCC |
| *RuDFR* | 5´AATCAGAAGAAGGTGAAGC | 5´CATTAKSACAAGTTTGGTG |
| *RuF3'5'H* | 5´ATGCCHCATGYYDCCTTAGCHAAAATGG | 5´TGGGCAATHGGRMGAGAYCC |
| *RuF3H* | 5´ATGGCTCCTACACCTACTAC | 5´TGGATCACCGTTCAACCTGTGGAAGG |
| *RuF3'H* | 5´CCTATCTCCAAGCTGTCATCAAG | 5´GTGGTATCCGTTGATTTCACAAC |
| *RuFLS* | 5´CCTACAGGGAAGTCAATGAGAAA | 5´CACATGGGATTTCAGTACCTTCT |
| *RuGST1* | 5´TACTAGAATCACAAGCACCAGCA | 5´ACCCAAAACTCACATAGACAACG |
| *RuGST2* | 5´GAACTCATTGCTTGAGAGCGTAG | 5´GATCTTCCACACTTCCTCTACCA |
| *RuLAR* | 5´GTGGAGTCCCATACACGTACATT | 5´CTGAAACTGATCTAACGGTGGAA |
| *RuPAL1* | 5´GAGGAACTGGGGACTAGTTTGTT | 5´AGCAGAGGATCAATCAGCTTTC |
| *RuPAL2* | 5´GACTTGCTCTTGTTAATGGCACT | 5´GAAAATCGCAGACAAGATTTCG |
| *RuMYB1* | 5´CTCATTGACAGGAACAGGTGTC | 5´CCTACAACAACACCAACGAGAAT |
| *RuMYB3* | 5´GAGCTGTAGGTTAAGATGGACAAA | 5´GTTTCCAAGGATAGAATGGAGATG |
| *RuMYB4* | 5´ACAGCTCAGGACTCTGCTACAAC | 5´GGTTTATAGACTCTTTGCCCACA |
| *RuMYB5* | 5´ACTCAATCCAGACTCCTCATCTG | 5´AGGAAGTGATTGGACTTTTAGGG |
| *RuMYB6* | 5´TCCTATGGAGTACTTCCAAGCTC | 5´TATGGCTGTTTAGTCCTCCTTGA |
