# Supplementary material for: Elicitation with Bacillus QV15 reveals a pivotal role of F3H on flavonoid metabolism improving adaptation to biotic stress in blackberry
Source: PLoS One. 2020 May 6;15(5):e0232626. doi: 10.1371/journal.pone.0232626 (PMC7202615; doi:10.1371/journal.pone.0232626)
Supplement: Attachment — Submitted filename: PONE-D-19-31790_reviewer.pdf [file pone.0232626.s009.pdf]

PLOS ONE

Elicitation with Bacillus QV15 reveals a pivotal role of F3H on flavonoid metabolism improving adaptation to biotic stress in blackberry

--Manuscript Draft--

**Manuscript Number:**	PONE-D-19-31790
**Article Type:**	Research Article
**Full Title:**	Elicitation with Bacillus QV15 reveals a pivotal role of F3H on flavonoid metabolism improving adaptation to biotic stress in blackberry
**Short Title:**	F3H plays a pivotal role on flavonoid metabolism in blackberry
**Corresponding Author:**	Beatriz Ramos-Solano<br>Universidad CEU San Pablo<br>Boadilla del Monte, Madrid SPAIN
**Keywords:**	Blackberry; flavonol-anthocyanin pathway; Bacillus amyloliquefaciens; flavonol-3-hydroxylase; PGPR; plant adaptation; biotic stress; photosynthetic pigments; elicitation
**Abstract:**	The aim of this study is to determine the involvement of the flavonol-anthocyanin pathway on plant adaptation to biotic stress using the Bacillus amyloliquefaciens QV15 to trigger blackberry metabolism and identify target genes to improve plant fitness and fruit quality. To achieve this goal, field-grown blackberries were root-inoculated with QV15 along its growth cycle. At fruiting, a transcriptomic analysis by RNA-Seq was performed on leaves and fruits of treated and non-treated field-grown blackberries after a sustained mildew outbreak; expression of the regulating and core genes of the Flavonol-Anthocyanin pathway were analysed by qPCR and metabolomic profiles by UHPLC/ESI-qTOF-MS; plant protection was found to be up to 88%. Overexpression of step-controlling genes in leaves and fruits, associated to lower concentration of flavonols and anthocyanins in QV15-treated plants, together with a higher protection suggest a phytoanticipin role for flavonols in blackberry; kempferol-3-rutinoside concentration was strikingly high. Overexpression of RuF3H (Flavonol-3-hidroxylase) suggests a pivotal role in the coordination of committing steps in this pathway, controlling carbon flux towards the different sinks. Furthermore, this C demand is supported by an activation of the photosynthetic machinery, and boosted by a coordinated control of ROS into a sub-lethal range, and associated to enhanced protection to biotic stress
**Order of Authors:**	Enrique Gutierrez-Albanchez
	Ana Gradillas
	Antonia García
	Ana Garcia-Villaraco
	Francisco Javier Gutierrez-Mañero
	Beatriz Ramos-Solano
**Additional Information:**	
**Question**	**Response**
**Financial Disclosure**<br><br>Enter a financial disclosure statement that describes the sources of funding for the work included in this submission. Review the submission guidelines for detailed requirements. View published research articles from *PLOS ONE* for specific examples.	This work was supported by grants from the Spanish Ministerio de Economía y Competitividad for projects AGL2013-45189 (BRS, JGM), CTQ2014-55279-R (AG) and grant reference BES-2014-0769990 to EGA.<br><br>The funders had no role in study design, data collection and analysis, decision to publish, or preparation of the manuscript

Powered by Editorial Manager® and ProduXion Manager® from Aries Systems Corporation

This statement is required for submission and **will appear in the published article** if the submission is accepted. Please make sure it is accurate.<br><br>**Unfunded studies**<br>Enter: *The author(s) received no specific funding for this work.*<br><br>**Funded studies**<br>Enter a statement with the following details:<br>• Initials of the authors who received each award<br>• Grant numbers awarded to each author<br>• The full name of each funder<br>• URL of each funder website<br>• Did the sponsors or funders play any role in the study design, data collection and analysis, decision to publish, or preparation of the manuscript?<br>• **NO** - Include this sentence at the end of your statement: *The funders had no role in study design, data collection and analysis, decision to publish, or preparation of the manuscript.*<br>• **YES** - Specify the role(s) played.<br><br>* typeset	
**Competing Interests**<br><br>Use the instructions below to enter a competing interest statement for this submission. On behalf of all authors, disclose any competing interests that could be perceived to bias this work—acknowledging all financial support and any other relevant financial or non-financial competing interests.<br><br>This statement **will appear in the published article** if the submission is accepted. Please make sure it is accurate. View published research articles from *PLOS ONE* for specific examples.	No authors have competing interests

Powered by Editorial Manager® and ProduXion Manager® from Aries Systems Corporation

**NO authors have competing interests**<br><br>Enter: *The authors have declared that no competing interests exist.*<br><br>**Authors with competing interests**<br><br>Enter competing interest details beginning with this statement:<br><br>*I have read the journal's policy and the authors of this manuscript have the following competing interests: [insert competing interests here]*<br><br>* typeset	
**Ethics Statement**<br><br>Enter an ethics statement for this submission. This statement is required if the study involved:<br><br>• Human participants<br>• Human specimens or tissue<br>• Vertebrate animals or cephalopods<br>• Vertebrate embryos or tissues<br>• Field research<br><br>Write "N/A" if the submission does not require an ethics statement.<br><br>General guidance is provided below. Consult the submission guidelines for detailed instructions. **Make sure that all information entered here is included in the Methods section of the manuscript.**	N/A

Powered by Editorial Manager® and ProduXion Manager® from Aries Systems Corporation

**Format for specific study types**<br><br>**Human Subject Research (involving human participants and/or tissue)**<br>• Give the name of the institutional review board or ethics committee that approved the study<br>• Include the approval number and/or a statement indicating approval of this research<br>• Indicate the form of consent obtained (written/oral) or the reason that consent was not obtained (e.g. the data were analyzed anonymously)<br><br>**Animal Research (involving vertebrate animals, embryos or tissues)**<br>• Provide the name of the Institutional Animal Care and Use Committee (IACUC) or other relevant ethics board that reviewed the study protocol, and indicate whether they approved this research or granted a formal waiver of ethical approval<br>• Include an approval number if one was obtained<br>• If the study involved *non-human primates*, add *additional details* about animal welfare and steps taken to ameliorate suffering<br>• If anesthesia, euthanasia, or any kind of animal sacrifice is part of the study, include briefly which substances and/or methods were applied<br><br>**Field Research**<br><br>Include the following details if this study involves the collection of plant, animal, or other materials from a natural setting:<br>• Field permit number<br>• Name of the institution or relevant body that granted permission	
**Data Availability**<br><br>Authors are required to make all data underlying the findings described fully available, without restriction, and from the time of publication. PLOS allows rare exceptions to address legal and ethical concerns. See the PLOS Data Policy and FAQ for detailed information.	Yes - all data are fully available without restriction

A Data Availability Statement describing where the data can be found is required at submission. Your answers to this question constitute the Data Availability Statement and **will be published in the article**, if accepted.<br><br>**Important:** Stating 'data available on request from the author' is not sufficient. If your data are only available upon request, select 'No' for the first question and explain your exceptional situation in the text box.<br><br>Do the authors confirm that all data underlying the findings described in their manuscript are fully available without restriction?	
**Describe where the data may be found in full sentences. If you are copying our sample text, replace any instances of XXX with the appropriate details.**<br><br>• If the data are **held or will be held in a public repository**, include URLs, accession numbers or DOIs. If this information will only be available after acceptance, indicate this by ticking the box below. For example: *All XXX files are available from the XXX database (accession number(s) XXX, XXX.)*.<br>• If the data are all contained **within the manuscript and/or Supporting Information files**, enter the following: *All relevant data are within the manuscript and its Supporting Information files.*<br>• If neither of these applies but you are able to provide **details of access elsewhere**, with or without limitations, please do so. For example:<br><br>*Data cannot be shared publicly because of [XXX]. Data are available from the XXX Institutional Data Access / Ethics Committee (contact via XXX) for researchers who meet the criteria for access to confidential data.*<br><br>*The data underlying the results presented in the study are available from (include the name of the third party*	All relevant data are within the manuscript and its Supporting Information files

and contact information or URL).<br>• This text is appropriate if the data are owned by a third party and authors do not have permission to share the data.<br><br>* typeset	
Additional data availability information:	

Powered by Editorial Manager® and ProduXion Manager® from Aries Systems Corporation

Manuscript Click here to download Manuscript Text file PLOS Ramos-Solano.docx

Elicitation with *Bacillus* QV15 reveals a pivotal role of F3H on flavonoid metabolism improving adaptation to biotic stress in blackberry

Enrique Gutiérrez Albanchez[1*], Ana Gradillas[2], Antonia García[2], Ana García-Villaraco[1], F. Javier Gutierrez-Mañero[1], Beatriz Ramos-Solano[1*].

[1]Plant Physiology, Pharmaceutical and Health Sciences Department, Faculty of Pharmacy, Universidad San Pablo-CEU, Madrid, Spain.

[2]Centre for Metabolomics and Bioanalysis, Chemistry and Biochemistry Department, Faculty of Pharmacy, Universidad San Pablo-CEU, Madrid, Spain

Universidad San Pablo CEU, Facultad de Farmacia, Carretera de Boadilla del Monte km. 5.3, 28668, Boadilla del Monte, Madrid, Spain

*Corresponding author

Email: bramsol@ceu.es (BRS)

Abstract

The aim of this study is to determine the involvement of the flavonol-anthocyanin pathway on plant adaptation to biotic stress using the *B.amyloliquefaciens* QV15 to trigger blackberry metabolism and identify target genes to improve plant fitness and fruit quality. To achieve this goal, field-grown blackberries were root-inoculated with QV15 along its growth cycle. At fruiting, a transcriptomic analysis by RNA-Seq was performed on leaves and fruits of treated and non-treated field-grown blackberries after a sustained mildew outbreak; expression of the regulating and core genes of the Flavonol-Anthocyanin pathway were analysed by qPCR and metabolomic profiles by UHPLC/ESI-qTOF-MS; plant protection was found to be up to 88%. Overexpression of step-controlling genes in leaves and fruits, associated to lower concentration of flavonols and anthocyanins in QV15-treated plants, together with a higher protection suggest a phytoanticipin role for flavonols in blackberry; kempferol-3-rutinoside concentration was strikingly high. Overexpression of *RuF3H* (Flavonol-3-hidroxylase) suggests a pivotal role in the coordination of committing steps in this pathway, controlling carbon flux towards the different sinks. Furthermore, this C demand is supported by an activation of the photosynthetic machinery, and boosted by a coordinated control of ROS into a sub-lethal range, and associated to enhanced protection to biotic stress.

Introduction

Rubus cv. Loch Ness is a plant that belongs to a large group of plants with beneficial properties for human health known as berries. This group is characterized for the high amount of secondary metabolites (flavonoids among others) present in their fruits, and in leaves [1, 2, 3]; benefits for human health relay on flavonoids to a great extent [4-5].

Plants have successfully colonized all environments of our planet, thanks to their ability to develop a plant-specialized metabolism as a part of their evolutionary process, which enables them to adapt to the continuous changing conditions along their lifetime [6]. Plant secondary metabolism confers plasticity to plants so that they are able to adapt to changing environmental conditions, usually adverse conditions, ensuring plant survival [7-8]. Hence, this metabolism is sensitive to different factors among which are biotic agents, like beneficial or harmful microorganisms, which can be used to trigger plant metabolism. Therefore, beneficial microorganisms constitute a biotechnological tool to improve plant fitness and enhance secondary metabolites contents in plant organs [9,10,11]. More precisely, the use of beneficial bacteria to trigger secondary metabolism involved in plant defense is gaining a lot of interest and there is increasing evidence of their effectiveness under controlled and field conditions to support their effects [12]. Furthermore, elicitation can be used as a tool to identify target genes to be edited by CRISPR/Cas9 with the aim to improve plant fitness and/or food quality [13].

Plant Growth Promoting Bacteria (PGPB) are beneficial strains naturally present in the rhizosphere of plants contributing to plant health. As it has been demonstrated certain strains trigger expression of some plant genes, defense related genes among others, enhancing plant defense metabolism; so when the pathogen tries to invade the

plant, it is already prepared and not dramatically infected [14, 15]. Therefore, PGPB appear as an alternative to chemical pesticides as well as tools to study plant metabolism. As pests in the agricultural systems are an important threat because they reduce plant yield and fruit quality, with the consequent economic losses, pest control is an unquestionable challenge for agriculture and to achieve food security, a term which refers to “food availability, in sufficient quantities with proper amount of nutrients and on a consistent basis”. Hence, finding effective biological agents is a challenge, and unraveling plant changes upon delivery of the biological will set the bases for a successful agronomic management.

The present study focuses on flavonoid metabolism, as it is highly expressed in blackberry, and both leaves and fruits contain high flavonoid concentrations [16]. Flavonoids belong to a metabolic network that mediates on plant adaptation to environmental stress Flavonoids are a ubiquitous group of secondary metabolites key for adaptation and survival on earth life [17], participating in many different processes of plant physiology [4, 18, 19, 20, 21, 22, 23, 6, 23]. Hence, a deeper knowledge of flavonoid metabolism and key enzymes controlling relevant branching points will allow us to manipulate plant metabolism in our benefit, for example in agriculture. Upon biotic stress challenge, they may play a different role in defense, as they can either be defensive molecules by themselves, behaving as phytoalexins, or they may be accumulated as phytoanticipins and transformed into the real phytoalexins upon pathogen challenge [24-25].

Based on this background, and using *B.amyloliquefaciens* QV15 as a tool to trigger plant metabolism, the present study reports the systemic effects of root inoculated bacteria on blackberry leaves and fruits at the transcriptomic and metabolomic level, focusing on the flavonol-anthocyanin biosynthetic pathway (Fig 1).

The aim of this study was (i) to study the effects of elicitation with a beneficial biotic agent on blackberry leaf and fruit metabolism, (ii) to determine the involvement of the flavonol-anthocyanin pathway on plant adaptation to biotic stress. To achieve these objectives, a transcriptomic analysis by RNA-Seq was performed, and qPCR expression of the regulating and core genes of the Flavonol-Anthocyanin pathway and metabolomic changes by UHPLC/ESI-qTOF-MS analysis on inoculated and non-inoculated field-grown blackberries at fruiting, after a sustained mildew outbreak were determined.

Fig 1. Biosynthesis of anthocyanins, flavonols and catechins via the flavonoid pathway in *Rubus* cv. Loch Ness. Phenylalanine ammonio-lyase (RuPAL1 and RuPAL2), Cinammate-4-hydroxylase (RuC4H), 4-coumaryl-CoA ligase (Ru4CL), Chalcone synthase (RuCHS), Chalcone Isomerase1 (RuCHI1), Chalcone Isomerase2 (RuCHI2), Flavonol-3-hydroxylase (RuF3H), Flavonoid 3´5´hydroxylase (RuF3´5´H), Flavonoid 3´hydroxylase (RuF3’H), Flavonol synthase (RuFLS), Leucoanthocyanidin reductase (RuLAR), Anthocyanidin reductase (RuANR), Dehydroflavonol reductase (RuDFR), Anthocyanidin synthase (RuANS), Flavonol and Anthocyanidin Glycosiltransferases (RuFLS and RuAGT).

Materials and methods

Bacterial strain

Bacillus amyloliquefaciens QV15 (CECT 9371) is a gram positive sporulated bacilli; it was isolated from the rhizosphere of *Pinus pinea* [26]. It produces siderophores and stimulates pine growth [27], enhances defence against *Pseudomonas syringae* (DC3000) and protects against abiotic stress (NaCl 60 mM) [28].

Bacterial strain was maintained at -80 ºC in nutrient broth with 20% glycerol. Inoculum was prepared by streaking strains from -80 ºC onto plate count agar (PCA) plates, incubating plates at 28 ºC for 24 h. After that, QV15 was transferred to Luria Broth liquid media (LB) that was grown under shaking (1000 rpm) at 28 ºC for 24 hours to obtain a $2x10^9$ cfu/mL inoculum.

Plant Materials and experimental set up

Rubus cv. Loch Ness is a high yielding tetraploid (4n = 28) blackberry, and one of the most widely cultivated varieties. In southwest Spain, blackberries are produced under "winter cycle" involving an artificial cold period in order to induce flowering upon transplant to greenhouses. Blackberry cycle has three stages: vegetative, flowering and flowering-fruiting; the duration of these stages is variable depending on the transplant moment, and each stage approximately accounts for one third of the plant's life.

The *Rubus c*v. Loch Ness plants used in this study were kindly provided by Agricola El Bosque S.L. (Lucena del Puerto, Huelva, Spain). Plants and greenhouses were handled according to regular agricultural practices [29]. Plants were grown in Huelva (South Western Spain) from September 2014 to February 2015 under "winter cycle". A total of 360 plants were in the trial, arranged in six greenhouses; each greenhouse had two lines with 60 plants each, being each line one replicate with 60 repetitions; 3 lines were inoculated and 3 lines were left as non-inoculated controls. QV15 was root inoculated every 15 days during the whole plant cycle with 0.5 L of inoculum at 10^7 cfu/ml per plant.

In this experiment, plants were transplanted at the end of September 2014, flowering took place in November and maximum fruiting in January 2015. Number of flowers per square meter at flowering, and accumulated fruit production, were recorded. A natural Mildew outbreak took place from November till harvest, and disease incidence was recorded by visual evaluation of the surface affected carried out by 3 independent expert observers. At fruiting, leaves were sampled and immediately frozen in liquid nitrogen, and then brought to the lab. Three replicates were taken, being each one constituted from plant material of 60 plants; leaves were randomly sampled and pooled constituting one replicate and red fruits were taken at the same moment Samples were powdered with liquid nitrogen for RNA extraction for qPCR and further analysis: pigments (chlorophylls) and bioactives (phenols, flavonols, anthocyanins) determination by colorimetry and UHPLC/ESI-qTOF-MS

RNA extraction

Total RNA was isolated from each replicate with Plant/Fungi Total RNA Purification kit [30] (NORGENTM) (DNase treatment included) and, a reverse transcription followed by a RT-qPCR and RNA-Seq were performed.

RNA-Seq

Quality control and Library preparation

RNA was obtained from the three biological replicates for RNA-Seq analysis. RNA samples were DNase treated and extracted as described before. Thirty µl of RNA samples were passed through quality control with Nanodrop™ and Experion™, after

that total RNA meeting quality criteria was sent to Exiqon™ for sequencing. A total of three libraries were done for each organ

During the library preparation, poly-A tailed transcripts were enriched, as mRNA sequencing targets this type of transcripts, using an Oligo-dT magnetic bead-based system were enriched. The poly-A tailed transcripts include the coding mRNAs (1-4% of the whole transcriptome), so by this enrichment the appropriate depth of the sequencing for coding mRNA was achieved. The library preparation also retains information of which of the two strands of DNA was used to transcribe the given RNA, which enables the detection of antisense transcript expression. Mitochondrial poly-A tailed transcripts were bioinformatically filtered since they were considered to be high abundance sequences. The sequencing was paired end, which increases the mapping percentage to poorly annotated genomes, and identifies splice variants.

Two types of sequencing library quality controls were performed, firstly after the library preparation and bead based size selection, the size distribution of the library was evaluated using a Bioanalyzer high sensitivity DNA chip. Then, qPCR based quantification of each library was performed, and samples were normalized and pooled in equimolar ratios. After pooling of sample libraries, qPCR based quantification was performed on the library pool to ensure optimal concentration for cluster generation on the flow cell.

Sequencing

The library pools to be sequenced were denatured and diluted/neutralized in the required concentrations. Then, cluster generation was performed on the appropriate flow cell using single molecule clonal amplification. Finally, the high- throughput next

generation sequencing was performed using the Illumina sequencing technology platform.

RNA library assembly

Ribosomal RNA removal was performed with the Ribo-Zero rRNA kit removal kit. Generation of libraries was performed with the TruSeq Stranded Total RNA library Prep kit following manufacturer's recommendations. We started from 2 μg of total RNA (RIN>9) libraries, which were sequenced using a HiSeq2500 instrument (Illumina Inc, San Diego, CA, USA). Sequencing readings were paired end with a length of 101bp reading performed in 6 samples. The estimated coverage was around 52 million reads per sample (1 lane). Library generation and RNA sequencing was done at Sistemas Genómicos S.L. (Valencia, Spain) following manufacturer´s instructions.

RNA transcriptomic analysis

The quality control of the raw data was performed using the FastQC v0.11.4 tool. Then, the raw paired-end reads were mapped against the *Rubus occidentalis* genome v1.1 provided by GDR database using Tophat2 2.1.0 algorithm [31]. Insufficient quality reads (phred score<5) were eliminated using Samtools 1.2 [32] and Picard Tools 2.12.1. In this step, we assessed the GC distribution (i.e. the proportion of guanine and cytosine bp along the reads), which should have a desired distribution between 40–60%. Second, distributions of duplicates (quality of sequencing indicator) were evaluated to confirm that our sequencing contained small proportion of duplicates. Expression levels were calculated using the HiTSeq [33]. This method employs unique reads for the estimation of gene expression and filters the multimapped reads. Differential expression analysis between conditions was assessed using DESeq2 [34].

Finally, we selected differentially expressed genes with a P value adjusted by FDR [35] 0.05 and a fold change of at least 1.2. The DEG analysis between groups was done using statistical packages designed by Python and R. Using DESeq2 algorithm [34] applying a differential negative binomial distribution for the statistics significance [31-34] we identified genes differentially expressed. We considered as differently expressed genes those with a FC value below −1.2 or higher than 1.2 and with P value (Padj) corrected by FDR≤0,05 to avoid identification of false positives across the differential expression data.

Photosynthetic pigments (chlorophylls and carotenoids) extraction and quantification in leaves

Chlorophylls were isolated from each replicate. One hundred mg of powdered leaves were dissolved in 3 mL of acetone 80%, vortexed and centrifuged for 5 min at 10000 r.p.m (Hermle Z233 M-2). Absorbance was measured at 645, 662 and 470 nm in a Biomate 5 spectrophotometer. To calculate chlorophyll a, chlorophyll b, and carotenoids, the following formulas were used [36].

- Chl a (mg g^{-1}) = [(12.25 x Abs_{663}) - (2.79 x Abs_{647})] x V (ml)/ weigh (mg).
- Chl b (mg g^{-1}) = [(21.5 x Abs_{647}) - (5.1 x Abs_{663})] x V (ml)/ weigh (mg).
- Carotenoids (mg g^{-1}) = ((1000 x Abs_{470}) – (1.82 x Chl a)- (85.02 x Chl b))/198) x V (ml)/ weigh (mg).

Bioactive characterization

Colorimetric quantification

Two extracts were prepared. One g of powdered leaves from each replicate was mixed with 9 mL of methanol 80% for phenols and flavonols determination, and one gram with 9 mL of methanol 80% 0.1%HCl for anthocyanins determination; then, samples were sonicated for 10 min and centrifuged for 5 min at 5000 rpm. Supernatants were frozen and lyophilized. The same was done with fruit samples

Total phenols were quantitatively determined with Folin-Ciocalteu reagent (Sigma. Aldrich, St Louis, MO) by a colorimetric method described in [37], with some modifications; gallic acid was used as standard (Sigma-Aldrich, St Louis, MO). One milliliter of extract was mixed with 0.250 mL of Folin-Ciocalteu 2N (Sigma. Aldrich, St Louis, MO) and 0.75 mL of Na_2CO_3 20% solution. After 30 minutes at room temperature, absorbance was measured at 760 nm with an UV-Visible spectrophotometer (Biomate 5). A gallic acid calibration curve was made (r= 0.99). Results are expressed in mg of gallic acid equivalents per g of powdered leaves. The same was done with fruit samples. All samples were measured in triplicate.

Total flavonols were quantitatively determined through the test described in [38], using catechin as standard (Sigma-Aldrich, St Louis, MO). One milliliter of extract was added to a 10 mL flask with 4 mL of distilled water. After that, 0.3 mL of $NaNO_2$ 5%, and 0.3 mL of $AlCl_3$ 10% were added after 5 minutes. One minute later, 2 mL of NaOH 1 M were added and the mixture was brought to 10 mL with distilled water. The solution was mixed and measured at 510 nm with an UV-Visible spectrophotometer (Biomate 5). A catechin calibration curve was made (r= 0.99). Results are expressed as mg of catechin equivalents per g of powdered leaves. The same was done with fruits. All samples were measured in triplicate.

Total anthocyanins were quantitatively determined through the pH differential method described by [39]. Extracts were diluted in pH 1 buffer (0.2 M KCl) and pH 4.5 (1M CH_3COONa) in 1:15 proportion. After that, absorbance was measured at 520 and 720 nm respectively, in a UV-Visible spectrophotometer (Biomate 5). A cyanidin-3-glucoside calibration curve was made (r= 0.99). Results are expressed in cyanidin-3-glucoside equivalents per g of powdered leaves. The same was done with fruits. All samples were measured in triplicate.

UHPLC/ESI-qTOF-MS Phenolics and Flavonoids Analysis

Standards and solvents: Phenolic acids including, gallic acid, caffeic acid, ferulic acid and chlorogenic acid were purchased from Sigma (St. Louis, MO, USA) and flavonoids including, kaempferol, kempherol-3-*O*-rutinoside, kempherol-3-*O*-glucoside, quercetin, quercetin-3-*O*-rutinoside, quercetin-3-*O*-glucoside, (+)-catechin, (-)-epicatechin and cyanidin-3-*O*-glucoside, were purchased from Sigma and from Extrasynthese Co. ™ (Geney, France).

The standard solutions (10 ppm) were prepared in methanol. All the solvents, as methanol and acetonitrile (*Honeywell Riedel-de Haen*), were LC-MS grade. Purified water was obtained from Milli-Q Plus™ System from Millipore (Milford, MA, USA). Formic acid was purchased from Aldrich (St. Louis, MO, USA)

Sample preparation

The extraction of phenolics was performed as follows: 30 mg of powder were added to 300 µL of methanol. The mixture was vortexed for 2 min, sonicated for 5 min and centrifuged at 3.500 rpm for 5 min at 4 ºC. The supernatants were then collected and stored at -20 ºC until use for LC/MS analysis.

UHPLC/ESI-qTOF-MS Analysis

Samples were analyzed on a 1290 Infinity series UHPLC system coupled through an electrospray ionization source (ESI) with Jet Stream technology to a 6550 iFunnel QTOF/MS system (Agilent Technologies, Waldbronn, Germany) as described in [40].

For the separation, a volume of 2 µL was injected in a reversed-phase column (Zorbax Eclipse XDB-C18 4.6 × 50 mm, 1.8 µm, Agilent Technologies) at 40 °C. The flow rate was 0.5 mL/min with a mobile phase consisted of solvent A: 0.1% FA, and solvent B: methanol. Gradient elution consisted of 2% B (0-6 min), 2-50% B (6-10 min), 50-95% B (11-18 min), 95% B for 2 min (18-20 min), and returned to starting conditions 2% B in one minute (20-21 min) to finally keep the re-equilibration with a total analysis time of 25 min.

Detector was operated in full scan mode (*m/z* 50 to 2000), at a scan rate of 1 scan/s. Accurate mass measurement was assured through an automated calibrator delivery system that continuously introduced a reference solution, containing masses of *m/z* 121.0509 (purine) and m/z 922.0098 (HP-921) in positive ESI mode; whereas *m/z* 112.9856 (TFA) and m/z 922.009798 (HP-921) in negative ESI mode. The capillary voltage was ±4000 V for positive and negative ionization mode. The source temperature was 225 °C. The nebulizer and gas flow rate were 35 psig and 11 L/min respectively, fragmentor voltage to 75V and a radiofrequency voltage in the octopole (OCT RF Vpp) of 750 V.

For the study, MassHunter Workstation Software LC/MS Data Acquisition version B.07.00 (Agilent Technologies) was used for control and acquisition of all data obtained with UHPLC/ESI-qTOF-MS.

For quantification, each standard was injected twice in four different concentrations to build up callibration curves in which sample peak areas were interpolated.

Data treatment

UHPLC-MS data processing was performed by MassHunter Qualitative Analysis (Agilent Technologies) Software version B.08.00 using Molecular Feature Extraction (MFE).

RT-qPCR Analysis

The retrotranscription was performed using iScript tm cDNA Synthesis Kit (Bio-Rad). All retrotranscriptions were performed using a GeneAmp PCR System 2700 (Applied Biosystems): 5 min 25 ºC, 30 min 42 ºC, 5 min 85 ºC, and hold at 4 ºC. The amplification were performed with a MiniOpticon Real Time PCR System (Bio-Rad): 3 min at 95 ºC and then 39 cycles consisting of 15 s at 95 ºC, 30 s at 55 ºC and 30 s at 72 ºC, followed by melting curve to check the results. To describe the expression obtained in the analysis, cycle threshold (Ct) was used. Standard curves were calculated for each gene, and the efficiency values ranged between 90 and 110%. *HISTONE H3* (*HIS*) reference gene was used. Results for gene expression were expressed as differential expression by the $2^{-\Delta\Delta Ct}$ method (62). Control expression is set at 1, therefore only increases above one are considered. Core and regulatory genes studied and the primers used for each appear in Table 3 of supplementary material.

Statistical analysis

To evaluate treatment effects on photosynthetic pigments, bioactive contents and gene expression, one-way ANOVA analysis were performed. When significant differences appeared ($p<0.05$), LSD test (Least significant Difference) from Fisher was used. Statgraphics plus 5.1 for Windows was the program used.

Results

Evaluation of plant fitness

There is a significant increase of flowers per square meter in QV15-inoculated plants not associated to an increase in production. The Mildew outbreak started in November and was maintained throughout the plant cycle; controls showed 15% affected surface on average, while QV15 treated plants showed an average 5%. The relative disease index determined at fruiting indicates a rough 88% protection against the natural fungal disease (table 1).

Table 1. Plant fitness parametres in controls and QV15 treated plants.

	**Flowers/m2**	**Production (Kg)**	**Evolution of disease (%affected surface)**	**Relative disease index (%)**
**Control**	237.95 ± 2.28 (a)	6.2 ± 0.22 (a)	15% (b)	100 ± 1.05 (b)
**QV15**	323.5 ± 1.77 (b)	6.4 ± 0.09 (a)	5% (a)	12.02 ± 0.36 (a)

Number of flowers per square meter of blackberry plants at flowering. Production (Kilograms per plant). Disease incidence measured as affected leaf surface (%) with Mildew symptoms in blackberry plants from November (21st November) to harvest in february (02/05/2018). Relative disease index expressed as accumulated values of

affected leaf surface (%) with Mildew symptoms relative to controls. Different letters denote statistically significant differences according to LSD test (p<0.05).

RNA-Seq

In a typical experiment of whole transcriptome analysis, the number of mapped lectures to the reference genome is around 50%. In this case, in which the reference genome is *Rubus occidentalis* (v1.0 &Annotation v1 from the database of GDR), the mapping fraction obtained was around 50% (49.97% to 52.43%) (Table S1, supplementary material).

After sequencing and mapping alignment, normalized and differential expression (Control vs. QV15), a total of 29,126 genes were identified in leaves. The heatmap diagram (figure 2) shows the result of the two-way hierarchical clustering of RNA transcripts and samples; it includes the 50 genes that have the largest coefficient of variation based on FPKM counts. Each row represents one gene and each column represents one sample. The color represents the relative expression level of a transcript across all samples. The color scale is shown below: red represents an expression level above the mean; green represents an expression level below the mean.

Fig 2. Heat Map and unsupervised hierarchical clustering by sample; top 50 genes with the largest coefficient of variation based on FPKM counts, a) in leaves b) in fruits

When leaf samples were compared, the expression pattern showed that 28,586 genes were equally expressed in both treatments (expression without significant differences), 173 genes were significantly overexpressed in leaves of controls, and 367 genes were significantly overexpressed in leaves of QV15-treated plants (figure 3a). When fruits were compared, expression of 27,866 genes showed non-significant

differences, while genes overexpressed in controls accounted for 595, and genes overexpressed in QV15-treated plants accounted for 664 (figure 3b), being these numbers triple and double than in leaves, respectively.

Fig 3 Venn diagram of overexpressed and common genes in blackberry leaves (a) and fruits (b) from control and QV15-treated plants

Overexpressed genes in leaves appear in file1 (supplementary material). In controls, most genes are related to the phenylpropanoids-flavonoid pathway, and sugar metabolism. Overexpressed genes in leaves of QV15-treated plants are related to an active photosynthesis (mostly related to photosystems I and II), to an active regeneration of photosystems including pigments biosynthesis, and to an efficient capacity of ROS scavenging, as shown by the high number of transcripts of superoxide dismutase (SOD), and ascorbate peroxidase (APX). It is worth mentioning the high expression of glutathione-S-transferase 2 (GST2) (Table S2. supplementary material).

Two groups appear among overexpressed genes in control fruits (file S2, supplementary material); in one hand, a high vacuolar activity and an active sucrose metabolism are enhanced, and in the other hand, many transcripts of ubiquitin-protein ligases, serin/theronin kinases and Fbox/FBD/LRRs. In QV15-treated fruits, specialized defense enzymes such as subtilisin, different glucanases and chitinases, and a striking overexpression of GDSL esterase/lipases, a family of proteins that has been related to secondary metabolites synthesis and plant defense [47].

Photosynthetic pigments (chlorophylls and carotenoids)

Chlorophylls and carotenoids were more abundant in leaves of QV15-treated plants (table 2). Control plants had 0.57 mg g^{-1} Chl a, 0.23 mg g^{-1} Chl b, and 0.38 mg g^{-1}

carotenoids; while QV15 treated plants had 0.72 mg g^{-1} Chl a, 0.36 mg g^{-1} Chl b, and 0.42 mg g^{-1} carotenoids; this represents a rough 54% increase in chlorophyll contents, mainly chlorophyll b, and 5% carotenoid content increase in QV15 treated plants.

Table 2. Leaf pigments in blackberry leaves in controls and QV15 treated plants.

Samples	Chlorophyll A (mg g^{-1})	Chlorophyll B (mg g^{-1})	Carotenoids (mg g^{-1})
**Control**	0.57 ± 0.007 (a)	0.23± 0.006 (a)	0.38± 0.004 (a)
**QV15**	0.72± 0.004 (b)	0.36± 0.017 (b)	0.42± 0.002 (b)

Quantification of chlorophyll A, B, and Carotenoids in blackberry leaves. Values are the average of 3 replicates ± SD. Different letters denote statistically significant differences between treatments for each parameter according to LSD test ($p<0.05$).

Phenolics and Flavonoids Characterization

Leaves of QV15 treated plants had lower values of total phenolics (-18%), total flavonoids (-33%) and total anthocyanins (-21%) than controls. Total phenolic contents averaged 16.88 mg g^{-1} and 13.69 mg g^{-1} for control and QV15 treated plants, respectively. Total flavonols represent between 11 and 9 % of total phenolics, with 1.8561 mg g^{-1} and 1.2463 mg g^{-1} for controls and QV15 treated plants, respectively. Total anthocyanins represent around 1.6% of total phenolics, with 0.284 mg g^{-1} and 0.2209 mg g^{-1} for controls and QV15 treated plants, respectively (Table 3).

Table 3. Leaf and fruit Bioactives in controls and QV15 treated plants.

Samples	Phenols ($mg\ g^{-1}$)	Flavonols ($mg\ g^{-1}$)	Anthocyanins ($mg\ g^{-1}$)
**Control Leaves**	16.88 ± 0.292 (b)	1.85 ± 0.061 (b)	0.28 ± 0.007 (b)
**QV15 Leaves**	13.69 ± 0.621 (a)	1.24 ± 0.078 (a)	0.22 ± 0.026 (a)
**Control Fruit**	5.04 ± 0.074 (x)	0.31 ± 0.012 (x)	0.64 ± 0.015 (x)
**QV15 Fruit**	4.41 ± 0.187 (y)	0.29 ± 0.003 (x)	0.68 ± 0.004 (y)

Quantification of total phenolics, flavonols and anthocyanins in blackberry leaves and (b) fruits. Values are the average of 3 replicates ± SD. Different letters denote statistically significant differences between control and treated leaves (a,b) or fruits (x,y), according to LSD test ($p<0.05$).

Fruits showed significantly higher levels in controls (5.6 mg/g) than QV15-treated plants (4.93mg/g), while total flavonoids and anthocyanins showed non-significant differences (Table 3).

Characterization of the methanolic extract of Blackberry leaves and fruits using UHPLC/ESI-qTOF-MS provided a good separation profile (Figure 4). The visualization of both chromatograms profile, run of 25 min, revealed more intense and well-resolved chromatographic peaks in negative compared to the positive ion mode.

Fig 4. Overlaid Chromatograms (positive and negative ion mode) obtained from LC/MS/TOFF analysis of the methanolic extract of BlackBerry leaf samples. Control samples are represented in Green while QV15 samples appear in red

This method allowed separation of three groups of compounds: phenolic compounds eluted first, from min 0,5 to min 13; then, ursane-type triterpene saponins, from 12,5 to 18,5 minutes, and chlorophyll break down products from 18,5 to 22 min (Fig 4). Chlorophyll breakdown products were higher in controls than in QV15 treated

plants. When fruits were analyzed, phenolic compounds eluted first; ursanes and chlorophyll breakdown products were not present (supplementary material, Figure S1)

In Blackberry leaves, characteristic flavonols were kaempferol and quercetin derivatives, and (−)-epicatechin among catechols, being gallic acid the most abundant phenolic acid. Table 4 shows that the most abundant flavonols identified in leaves were quercetin-3-O-rutinoside, kempferol-O-glucoside, quercetin-3-O-glucoside and kaempferol-3-O-rutinoside, being quercetin 3-O-glucoside the less abundant and kaempferol-3-O-rutinoside the most abundant.

Table 4. Identification and quantification of phenolic compounds in leaf samples of controls and QV15 treated plants.

**Peak No.**	**Compounds**	**t_R (min)**	**Molecular Formula**	**Monoisotopic Mass**	***m/z* experimental[b]**	**Area average (control)**	**µg/g**	**Area average (QV15)**	**µg/g**
**1**	gallic acid	3.0	$C_7H_6O_5$	170.0215	$[M-H]^-$= 169.0149	3.70E+05	9.143	3.52E+05	8.237
**2**	gentisic acid	8.3	$C_7H_6O_4$	154.0266	$[M-H]^-$ = 153.0196	1.14E+05	3.570	8.73E+04	2.740
**3**	6,7-dyhidroxycoumarin	9.2	$C_9H_6O_4$	178.0266	$[M-H]^-$ = 177.0181	8.51E+04	<LoQ	1.38E+05	<LoQ
**4**	(-)-epicatechin	9.4	$C_{15}H_{14}O_6$	290.0790	$[M-H]^-$ = 289.0723	3.63E+06	6.793	7.18E+05 *	3.124
**5**	quercetin-3-*O*-glucoside	11.0	$C_{21}H_{20}O_{12}$	464.0955	$[M-H]^-$ = 463.0887	6.41E+06	7.045	6.49E+06	7.324
**6**	quercetin-3-*O*-rutinoside	11.1	$C_{27}H_{30}O_{16}$	610.1534	$[M-H]^-$ = 609.1494	1.08E+07	28.201	7.96E+06	17.827
**7**	kaempferol-3-*O*-glucoside	11.5	$C_{21}H_{20}O_{11}$	448.1006	$[M-H]^-$ = 447.0938	4.23E+06	9.806	3.81E+06	8.148

**8**	kaempferol-3-*O*-rutinoside	11.5	$C_7H_6O_3$	138.0317	$[M-H]^- = 593.1520$	4.11E+06	37.056	5.70E+06	58.119
**9**	luteolin	12.7	$C_{15}H_{10}O_6$	286.0477	$[M-H]^- = 285.0395$	8,27E+05	<LoQ	1,04E+06	<LoQ

Identification and quantification of predominant compounds, expressed in µg/g, of phenolic compounds in leaf samples. Data is the average of 3 samples, with two injections each. <LoQ: below limit of quantitation (LoQ)

All of them except for kaempferol-O-rutinoside were higher in controls than in QV15-treated blackberries, as well as phenolic acids; interestingly, kempferol-O-rutinoside concentration was over 50% higher, and a marked decrease (54%) in (-)-epicatechin was observed (table 4).

In Blackberry fruits, characteristic flavonols were epicatechin, catechin, and flavonols were represented by kaempferol and quercetin derivatives; vanillic acid was also present in relevant amounts (Table 5). Epicatechin was by far, the most abundant compounds in red fruits, 200 µg/g on average, while flavonols and anthocyanins were on the 5-10 µg/g range. The most abundant flavonols were quercetin 3-O-glucoside, quercetin-3-O-rutinoside, kaempferol-3-O-rutinoside, and kempferol-O-glucoside while the most relevant anthocyanins were cyanidin 3-O-glucoside and cyanidin 3-O-arabinoside; delphinidin was detected only in QV15 treated plants. All of them are described from higher to lower abundance. Controls showed higher concentrations of all compounds except for kempferol derivatives and quercetin-O-galactoside, as occured in leaves.

Table 5. **Identification and quantification of phenolic compounds in fruit samples of controls and QV15 treated plants**.

Nº	NAME COMPOUND	MW (g/mol)	RT (Q-TOF)	Chemical Formula	Monoisotopic Mass	Area average (control)	µg/g	Area average (QV15)	µg/g
1	Salicyclic acid	138,12	11,5	$C_7H_6O_3$	138,0317	1,00e+05	<LOQ	1,40e+05	<LOQ
2	Vanillic acid	168,15	9,2	$C_8H_8O_4$	168,1467	6,14E+04	11	6,96e+04	14
3	Chlorogenic acid	354,31	8,9	$C_{16}H_{18}O_9$	354,0951	6,71E+05	<LoQ	5,95e+05	<LoQ
4	Phlorizin	436,41	11,3	$C_{21}H_{24}O_{10}$	436,1369	1,11E+05	<LoQ	4,98e+04	<LoQ
5	(-)-epicatechin	290,27	9,4	$C_{15}H_{14}O_6$	290,0790	6,24E+07	231,197	5,79e+07	214,749
6	(+)-catechin	290,27	8,5	$C_{15}H_{14}O_6$	290,0790	1,49E+06	4,914	1,26e+06	4,142
7	Kaempferol-3-O-glucoside	448,38	11,5	$C_{21}H_{20}O_{11}$	448,1006	3,52E+05	0,761	4,00e+05	0,864
8	Kaempferol-3-O-rutinoside	594,52	11,5	$C_{27}H_{30}O_{15}$	594,1585	3,66E+05	1,825	4,84e+05	2,415
9	Quercetin	302,24	12,4	$C_{15}H_{10}O_7$	302,0459	6,35E+04	<LoQ	5,64e+04	<LoQ
10	Quercetin-3-O-glucoside	464,38	11,1	$C_{21}H_{20}O_{12}$	464,0955	2,35E+06	5,016	2,32e+06	3,244
11	Quercetin-3-O-rutinoside	610,52	11,0	$C_{27}H_{30}O_{16}$	610,1534	1,53E+06	3,104	1,52E+06	2,871
12	Quercetin-3-O-galactoside	464,3763	9,4	$C_{21}H_{20}O_{12}$	464,0955	6,22E+05	a	7,08E+05	a
13	Malvidin-3-O-galactoside	493,39	9,6	$C_{23}H_{25}O_{12}$	493,1346		<LoQ		<LoQ
14	Delphinidin	303,2436	9,1	$C_{15}H_{11}O_7$	303,0505	1,18E+06	a	1,35E+06	a
15	Cyanidin-3-O-arabinoside	419,3589	9,4	$C_{20}H_{19}O_{10}$	419,0978	3,52E+05	a	2,64E+05	a
16	Cyanidin-3-O-glucoside	448,3769	9,2	$C_{21}H_{20}O_{11}$	448,1006	4,32E+07	2959,344	3,90E+07	2672,706

Identification and quantification on phenolic compounds present in blackberry fruits of control and QV15 treated samples. Data is the average of 3 biological replicates, 2 injections each. a) no standard available for quantification. <LoQ: below limit of quantitation (LoQ)

In addition to those compounds, an exhaustive analysis of other peaks was carried out by comparing the full TOF mass spectral data features to a list of possible compounds showing that mass. Some interesting compounds with bioactive potential were identified such as procyanidins, or galactosyl-diacyl-glycerid derivatives and the

ellagic tannin sanguiin H6, which could be identified only in the negative mode. Interestingly, all were higher in controls except for a galactosyldiacy-glycerid, which appeared only in QV15 treated fruits, in the positive mode, showing a large area in the chromatogram (supplementary material figure 1s).

RT-qPCR Analysis of core and regulatory genes of the Flavonol-Anthocyanin Pathway

Figures 5 and 6 show differential expression of the regulatory and core genes of the flavonol-anthocyanin pathway in leaves and fruits, respectively. Control expression is marked as 1, therefore, expression values over one indicate overexpression in QV15 treated plants; conversely, values below one can be interpreted as overexpression in controls. Asterisks indicate statistically significant differences.

In leaves of QV15-inoculated plants, the transcription factors *RuMYB3* and *RuMYB5* were significantly overexpressed (insert Fig), while *RuMYB1* and *RuMYB4* were overexpressed in controls. In general, expression of the flavonol-anthocyanin pathway core genes was higher in controls (Fig 5). Two isoforms were studied for *RuPAL, RuCHI*, and *RuGST*. Both *RuPAL* isoforms were overexpressed in control plants, being overexpression of *RuPAL1* significantly higher in controls than in QV15 treated plants. *RuCHI1, RuFLS, RuLAR, RuANR, RuDFR* and *RuANS* were overexpressed in control plants. Last enzyme of phenylpropanoids, *Ru4CL,* and last of early flavonol biosynthetic genes, *RuF3H* , were significantly overexpressed in QV15 treated plants. Genes encoding for the other enzymes *RuC4H, RuCHS, RuCHI2, RuF3´5´H* and *RuF3´H* were similarly expressed in control and QV15 treated plants. It was also found that *RuGST1* (glutathione S transferase 1) was overexpressed in control

plants, while *RuGST2* (glutathione S transferase 2) was overexpressed in QV15 treated plants (supplementary material. table S2).

Fig 5. Flavonol-Anthocyanin Pathway gene expression analyzed by RT-qPCR in leaves. The line set at value of 1 represents gene expression in controls, so values over one indicate overexpression in QV15 treated plants and values below one indicate overexpression in controls. Phenylalanine ammonio-lyase (*RuPAL1* and *RuPAL2*), Cinammate 4 hydroxylase (*RuC4H*), 4-coumaryl-CoA ligase (*Ru4CL*), Chalcone synthase (*RuCHS*), Chalcone Isomerase1 (*RuCHI1*), Chalcone Isomerase2 (*RuCHI2*), Flavonol-3-hydroxylase (*RuF3H*), Flavonoid 3´5´hydroxylase (*RuF3´5´H*), Flavonoid 3´hydroxylase (*RuF3'H*), Flavonol synthase (*RuFLS*), Leucoanthocyanidin reductase (*RuLAR*), Anthocyanidin reductase (*RuANR*), Dehydroflavonol reductase (*RuDFR*), Anthocyanidin synthase (*RuANS*). Insert: Flavonol-anthocyanin pathway regulatory genes. Asterisks indicate significant differences, according to Fisher test ($p<0.05$).

In fruits, expression of the flavonol-anthocyanin pathway core genes was higher in controls (Fig 6). Two isoforms were studied for *RuPAL*, *RuCHI*, and *RuGST*. Both *RuPAL* isoforms were overexpressed in control plants, being overexpression of *RuPAL2* significantly higher in controls than in QV15 treated plants. *Ru4CL*, *RuCHI1*, *RuCHI2* (early genes of flavonol-anthocyanin pathway), *RuF3´5´H*, *RuF3´H*, *RuFLS* (late genes of flavonol-anthocyanin pathway), and *RuANS* were overexpressed in control plants. *RuCHS, RuF3H* (early steps), *RuLAR,* (catechin pathway), and *RuDFR* (anthocyanin pathway) were significantly overexpressed in QV15 treated plants. It was also found that *RuGST2* (glutathione S transferase 1) was overexpressed in control plants, while *RuGST1* (glutathione S transferase 2) was overexpressed in QV15 treated plants (table S2). In QV15 inoculated plants, the transcription factor *RuMYB5* was significantly

overexpressed, while *RuMYB1*, *RuMYB3*, *RuMYB4* and *RuMYB6* were overexpressed in controls (insert, Fig 6).

Fig 6. Flavonol-Anthocyanin Pathway gene expression analyzed by RT-qPCR in fruits. The line set at value of 1 represents gene expression in controls, so values over one indicate overexpression in QV15 treated plants and values below one indicate overexpression in controls Phenylalanine ammonio-lyase (*RuPAL1* and *RuPAL2*), Cinammate 4 hydroxylase (*RuC4H*), 4-coumaryl-CoA ligase (*Ru4CL*), Chalcone synthase (*RuCHS*), Chalcone Isomerase1 (*RuCHI1*), Chalcone Isomerase2 (*RuCHI2*), Flavonol-3-hydroxylase (*RuF3H*), Flavonoid 3´5´hydroxylase (*RuF3´5´H*), Flavonoid 3´hydroxylase (*RuF3'H*), Flavonol synthase (*RuFLS*), Leucoanthocyanidin reductase (*RuLAR*), Anthocyanidin reductase (*RuANR*), Dehydroflavonol reductase (*RuDFR*), Anthocyanidin synthase (*RuANS*). Insert: Flavonol-anthocyanin pathway regulatory genes. Asterisks indicate significant differences, according to Fisher test ($p<0.05$).

Discussion

The results presented in this study indicate that QV15 triggers plant metabolism, improving plant fitness, adaptation to biotic stress and stimulating the flavonol-anthocyanin pathway in blackberry.

The responses triggered by this strain in the plant involves activation of gene expression related to photosynthesis and oxidative stress and specialized protective enzymes. The abundant transcripts related to photosynthesis found in leaves of QV15 treated plants reflect an active system for light reactions, an improvement in the efficiency of the photosynthetic electron transport chain, supported by overexpressed genes related to biosynthesis of photosynthetic pigments, mainly chlorophylls A and B.

This expression is consistent with the significantly higher levels in chlorophylls and carotenoids of QV15-treated plants (table 2), also reported for other *Bacillus* strains [41]. Furthermore, the UHPLC/ESI-qTOF-MS analysis indicated lower levels of chlorophyll breakdown products in elicited plants, so the positive effects on pigments could be explained by either an increased biosynthesis, or a decreased degradation, or both (Fig 4, table 2). The high activity of light reactions seems to be coordinated with an active carbon fixation, as overexpressed transcripts of ribulose bisphosphate carboxylase (RuBisCO) are found. Also consistent with the high activity of light reactions, abundant transcripts of the enzymatic pool of antioxidants were also observed and overexpressed in QV15 treated plants (supplementary material) suggesting a protective role against oxidative stress, and confirming enhanced plant fitness [42-43]. A striking overexpression of the isoenzyme glutathione-S-transferase (GST2), an enzyme with a strong protective role against oxidative stress, contributes to the enhanced plant fitness, as it is consistent with the high expression of the enzymatic pool of antioxidants. Furthermore, GST has been reported to be a molecular marker of induced resistance signaling mediated by ethylene in *A.thaliana* [44] and strongly related to phenylpropanoid-flavonoid transport within the plant [17].

Overexpressed genes in fruits, in one hand, reveal high vacuolar activity and an active sucrose metabolism, and in the other hand, the strong stress defense response and cell death is reflected in the many transcripts of ubiquitin-protein ligases, serin/theronin kinases and Fbox/FBD/LRRs [45] in controls, that in fact show a higher disease incidence. The F-box genes constitute one of the largest gene families in plants involved in degradation of cellular proteins. F-box proteins can recognize a wide array of substrates and regulate many important biological processes as among which are biotic and abiotic stress responses. Conversely, in QV15-treated fruits, defense response relies

on specialized defense enzymes such as subtilisin, glucanases and chitinases [46], and a striking overexpression of GDSL esterase/lipases, a family of proteins that has been related to secondary metabolites synthesis and plant defense [47]. This reveals the different pathways involved in protection and highlights the systemic response in QV15.

Stimulating the photosynthetic process suggests that the increase in the carbon fixed will be fed into growing leaves, flowers, and fruits, enhancing plant growth and probably increasing fruit yield, as reported for some beneficial bacterial strains [11-43]. This active metabolism provides a metabolic support to the high increase of flowers recorded which should be translated into a fruit yield increase. However, no significant increases in fruit yield were detected probably due to the Mildew outbreak after flowering, in which QV15 treated plants showed less disease symptoms than controls, with a protection that ranged between 87 to 68% along plant cycle (Table 1) [48]. That protection involves deviation of plant resources to plant defense, therefore compromising plant yield as balancing immunity and plant yield is the key for survival [49].

Our rationale was to demonstrate the role of flavonoids in adaptation to biotic stress, with a double aim, i) protection and ii) fruit quality. On one hand, to stimulate flavonoid synthesis on leaves to improve plant defense, as these secondary metabolites have been reported to play a relevant role in defense, being of great importance against biotic stress [51-52]. On the other hand, to benefit from this stimulation to enrich fruits on flavonoids and anthocyanin contents [52] as they are bioactive molecules good to prevent onset of disease [53]. More precisely, the flavonoids reported here, refer to the profile of total phenolics, flavonols and anthocyanins.

Flavonoids may alternatively play a role as phytoalexins or phytoanticipins, depending on the plant species, or even within different tissues of the same plant [25-54]. An increase upon pathogen challenge would indicate a role as phytoalexins, [55] while a decrease upon pathogen challenge would indicate a phytoanticipin role [24]. Moreover, the aglycons of flavonols have been reported to be more effective against fungi than their methyl derivatives [56-57] while flavanes, proanthocyanidins and isoflavones have been reported to be more effective against bacteria [58]. In control plants, flavonols were higher than in QV15 treated plants; this situation would indicate a role as phytoalexins in blackberry leaves, as flavonols increase in response to pathogen elicitors [54].

However, despite the lower flavonol concentration found in leaves of QV15 treated plants as well as that of total phenolics and anthocyanins (table 2), there was lower disease incidence. Consistent with the role of phytoanticipins, flavonols would be effectively transformed into another molecule, the phytoalexin, also resulting in lower flavonol levels in plant [54]. This statement is supported in part by the striking lower concentration in (-)-epicatechin and quercetin derivatives registered in QV15 treated leaves (table 4) and fruits (table 5), and overexpression of key genes in the pathway (Fig 5 and 6). Interestingly, an noticeable accumulation of kempferol-3-rutinoside was detected (tables 4 and 5) only in QV15 treated plants, suggesting a putative role in defense which is worth exploring since differential effects of each type of flavonol have been reported [59]. Irrespective of the fate of each molecule, the net balance of flavonol pool results in lower concentration in QV15 treated plants, which still remain more protected. As regards to fruits, no differences were found between controls and QV15-treated fruits in neither bioactive concentration, and still, Kempferol-3-rutinoside was strikingly high as in leaves, reinforcing the notion of a relevant role of this molecule.

Transcript profiling revealed coordinated increased transcript abundance for genes encoding enzymes of committing steps in the flavonol-anthocyanin pathway as well as in the regulators in QV15 treated plants, which was different in leaves and fruits. In leaves, only *Ru4CL* and *RuF3H*, the last enzymes in the phenylpropanoid pathway (*Ru4CL*), and last in the early flavonol-anthocyanin pathway (*RuF3H*), respectively, were overexpressed, suggesting a pivotal role for F3H in the control of the flavonol-anthocyanin pathway, consistent with the before mentioned metabolomic changes. The overexpression of key genes in the pathways ensures the carbon flux to that metabolic cluster, as enzymes involved in this pathway have been reported to cluster associated to the ER membrane for a better performance [60]. In fruits, overexpression of first and last gene of the early flavonol biosynthetic genes, first of anthocyanins and first for catechins revealed an active anthocyanin biosynthesis in QV15 fruits, anticipating the massive biosynthesis that is about to occur upon complete fruit maturation [52]. As flavonol and anthocyanin concentration is significantly lower in leaves of QV15 plants, together with a high concentration of GST transcripts, we hypotesize that leaf flavonols are being actively translocated to support anthocyanin synthesis in fruits (16). This process was more effective under the influence of QV15. Interestingly, the homologous to the positive regulators of late steps in the flavonol-anthocyanin pathway, *RuMYB3* and *RuMYB5* [61] were overexpressed in leaves, and only *RuMYB5* was in fruits, reinforcing the hypothesis of flavonoids being actively formed in leaves while leaf anthocyanin synthesis is inhibited and is activated in fruits. This suggests that *RuMYB3* could behave as anthocyanin repressor in blackberry, as the mode of control of the flavonoid pathway is quite specific of the species and moment of development [61]; furthermore, *RuMYB5* appears as the target for biotic stress adaptation used by this *Bacillus* strain.

Finally, the better performance in the inoculated plants against the pathogen could rely in other molecules, leaving a partial role in defense for flavonols, so the decrease would be due to translocation to fruits, to fulfill sink demand for anthocyanin biosynthesis, as they are vastly produced in leaves of blackberry plants [3]. Consistent with the partial role of flavonols in defense, the untargeted metabolomic profile revealed a characteristic series of triterpenoid pentacyclic saponins specific to the *Rubus* genus, ursanolic acids [40], which have been attributed to have an antimicrobial potential [62]; these compounds were more abundant in QV15 treated plants.

In summary, elicitation with QV15 has revealed a pivotal role of RuF3H controlling carbon fluxes towards the different sinks in the flavonol-anthocyanin pathway in blackberry and a relevant action of RuMYB5 in its control. The abundance of Kempferol-3-rutinoside leaves an open question about its role in defense. This C demand is supported by an activation of the photosynthetic machinery and boosted by a coordinated control of ROS into a sub-lethal range, in which GST2 seems to have a strong participation, and results in enhanced protection to biotic stress.

Acknowledgements

Authors thank for Agricola El Bosque S.L. “La Canastita” for providing help in blackberry crop

References

1.Thabti IW, Elfalleh N, Tlili M, Ziadi MG, Campos, Ferchichi A. Phenols, flavonoids and antioxidant and antibacterial activity of leaves and stem bark of *Morus* species. Intl J Food Prop. 2014;17(4):842-854. doi: 10.1080/10942912.2012.660722

2. Xi HF, Ma L, Wang LN, Li SH, Wang LJ. Differential response of the biosynthesis of resveratrols and flavonoids to UV-C irradiation in grape leaves. New Zeal J Crop Hortic Sci.2015; 43(3):163-172. doi: 10.1080/01140671.2014.989862.

3. Gutierrez E., García-Villaraco Velasco A, Lucas JA, Gutierrez-Mañero FJ, Ramos-Solano B. The flavonol-anthocyanin pathway in blackberry and arabidopsis: State of the art. In: Gonçalo C. Justino ed. Flavonoids - from biosynthesis to human health, InTech; 2017. p 129-150

4. Brunetti C, Di Ferdinando M, Fini A, Pollastri S, Tattini M. Flavonoids as antioxidants and developmental regulators: Relative significance in plants and humans. Intl J Molec Sci. 2013;14 (2): 3540-3555 doi: 10.3390/ijms14023540.

5. Cassidy A. Berry anthocyanin intake and cardiovascular health. Molec Aspects Med. 2017. doi: 10.1016/j.mam.2017.05.002.

6. Mouradov A, Spangenberg G. Flavonoids: a metabolic network mediating plants adaptation to their real estate. 2014 Front. Plant Sci. 5:16. doi: 10.3389/fpls.2014.00620

7. Oh MM, Trick HN, Rajashekara CB. Secondary metabolism and antioxidants are involved in environmental adaptation and stress tolerance in lettuce. J Plant Physiol. 2009;166(2):180-191. doi: 10.1016/j.jplph.2008.04.015.

8. Wink M. Plant secondary metabolism: Diversity, function and its evolution. Nat. Prod. Com. 2008;3(8):1205-1216.

9. Algar E, Gutierrez-Mañero FJ, Bonilla A, Lucas JA, Radzki W, Ramos-Solano B. Pseudomonas fluorescens N21.4 Metabolites Enhance Secondary Metabolism

Isoflavones in Soybean (Glycine max) Calli Cultures. J Agr Food Chem. 2012; 60(44): 11080-11087. doi: 10.1021/jf303334q.

10. Gutiérrez-Mañero FJ., García-Villaraco A, Lucas JA, Gutiérrez E, Ramos-Solano B. Inoculant / Elicitation Technology to Improve Bioactive/Phytoalexin Contents in Functional Foods. Int J Curr Microbiol Appl Sci 2012; 4(6):224-241.

11. Garcia-Seco D, Bonilla A, Algar E, Garcia-Villaraco A, Manero JG, Ramos-Solano B. Enhanced blackberry production using Pseudomonas fluorescens as elicitor. Agron Sust Devel. 2013; 33(2):385-392. doi: 10.1007/s13593-012-0103-z.

12. Shakya P, Marslin G, Siram K, Beerhues L, Franklin G. Elicitation as a tool to improve the profiles of high-value secondary metabolites and pharmacological properties of Hypericum perforatum. J Pharm Pharmacol. 2017.

13. Li XD, Wang YN, Chen S, Tian HQ, Fu DQ, Zhu BZ, Luo YB, Zhu HL. Lycopene Is Enriched in Tomato Fruit by CRISPR/Cas9-Mediated Multiplex Genome Editing. Front. Plant Sci. 2018;9. doi: 10.3389/fpls.2018.00559.

14. Bano A, Muqarab R. Plant defence induced by PGPR against Spodoptera litura in tomato (Solanum lycopersicum L.). Plant Biol. 2017; 19(3): 406-412. doi: 10.1111/plb.12535.

15. Selvaraj T, Rajeshkumar S, Nisha MC, Wondimu L, Tesso M. Effect of Glomus mosseae and plant growth promoting rhizomicroorganisms (PGPR's) on growth, nutrients and content of secondary metabolites in Begonia malabarica Lam. Maejo. Intl. J Sci Technol. 2008; 2 (3):516-525.

16. Gutierrez E, García-Villaraco A, Lucas JA, Gradillas A, Gutierrez-Mañero FJ, Ramos-Solano B. Transcriptomics, Targeted Metabolomics and Gene Expression of Blackberry Leaves and Fruits Indicate Flavonoid Metabolic Flux from Leaf to Red Fruit. Front Plant Sci. 2017b; 8:472.

17. Biała W, Jasin´ ski M. The Phenylpropanoid Case – It Is Transport That Matters. Front Plant Sci. 2018; 9:1610. doi: 10.3389/fpls.2018.01610.

18. Agati G, Brunetti C, Di Ferdinando M, Ferrini F, Pollastri S, Tattini M. Functional roles of flavonoids in photoprotection: New evidence, lessons from the past. Pl Phys Biochem. 2013;72:35-45. doi: 10.1016/j.plaphy.2013.03.014.

19. Bidart-Bouzat MG, Kliebenstein DJ. Differential levels of insect herbivory in the field associated with genotypic variation in glucosinolates in Arabidopsis thaliana. J Chem Ecol. 2008; 34 (8):1026-1037. doi: 10.1007/s10886-008-9498-z.

20. Cesco S, Mimmo T, Tonon G, Tomasi N, Pinton R, Terzano R, Neumann G, Weisskopf L, Renella G, Landi L, Nannipieri P. Plant-borne flavonoids released into the rhizosphere: impact on soil bio-activities related to plant nutrition. A review. Biol Fertil Soils 2012; 48(2):123-149. doi: 10.1007/s00374-011-0653-2.

21. Falcone Ferreyra ML, Rius SP, Casati P. Flavonoids: biosynthesis, biological functions, and biotechnological applications. Front Plant Sci. 2012; 3:222. doi: 10.3389/fpls.2012.00222.

22. Jiang N, Doseff AI, Grotewold E. Flavones: From Biosynthesis to Health Benefits. Plants 2016; 5(2). doi: 10.3390/plants5020027.

23. Weston LA, Mathesius U. Flavonoids: Their Structure, Biosynthesis and Role in the Rhizosphere, Including Allelopathy. J Chem Ecol. 2013; 39(2):283-297. doi: 10.1007/s10886-013-0248-5.

24. Algar E, Gutierrez-Manero FJ, Garcia-Villaraco A, Garcia-Seco D, Lucas JA, Ramos-Solano B. The role of isoflavone metabolism in plant protection depends on the rhizobacterial MAMP that triggers systemic resistance against Xanthomonas axonopodis pv. glycines in Glycine max (L.) Merr. cv. Osumi. Pl Phys Biochem. 2014; 82:9-16. DOI: 10.1016/j.plaphy.2014.05.001.

25. Boue SM, Cleveland TE, Carter-Wientjes C, Shih BY, Bhatnagar D, McLachlan JM, Burow ME. Phytoalexin-Enriched Functional Foods. J Agr Food Chem. 2009; 57 (7):2614-2622. doi: 10.1021/jf8040403.

26. Barriuso J, Pereyra MT, Garcia JAL, Megias M, Manero FJG, Ramos B. Screening for putative PGPR to improve establishment of the symbiosis Lactarius deliciosus-Pinus sp. Microb Ecol. 2005; 50(1):82-89. doi: 10.1007/s00248-004-0112-9.

27. Barriuso J, Solano BR, Manero FJG. Protection against pathogen and salt stress by four plant growth-promoting rhizobacteria isolated from Pinus sp on Arabidopsis thaliana. Phytopathol. 2008b; 98(6):666-672. doi: 10.1094/phyto-98-6-0666.

28. Barriuso J, Solano BR, Santamaria C, Daza A, Manero FJG. Effect of inoculation with putative plant growth-promoting rhizobacteria isolated from Pinus spp. on Pinus pinea growth, mycorrhization and rhizosphere microbial communities. J Appl Microbiol. 2008b; 105(5):1298-1309. doi: 10.1111/j.1365-2672.2008.03862.x.

29. Ramos-Solano B, Garcia-Villaraco A, Gutierrez-Manero FJ, Lucas JA, Bonilla A, Garcia-Seco D. Annual changes in bioactive contents and production in field-grown

blackberry after inoculation with Pseudomonas fluorescens. Plant Physiol Biochem 2014; 74:1-8. doi: 10.1016/j.plaphy.2013.10.029.

30. Kim D, Pertea G, Trapnell C, Pimentel H, Kelley R, Salzberg SL TopHat2: accurate alignment of transcriptomes in the presence of insertions, deletions and gene fusions. Genome Biol. 2013; 14(4). doi: 10.1186/gb-2013-14-4-r36.

31. Li H, Handsaker B, Wysoker A, Fennell T, Ruan J, Homer N, Marth G, Abecasis G, Durbin R. Proc Genome Project Data. The Sequence Alignment/Map format and SAMtools. Bioinformatics 2009; 25(16):2078-2079 doi: 10.1093/bioinformatics/btp352.

32. Anders S, Pyl PT, Huber W. HTSeq—a Python framework to work with high-throughput sequencing data. 2015; 31(2),166-169.

33. Love MI, Huber W, Anders S. Moderated estimation of fold change and dispersion for RNA-seq data with DESeq2. Genome Biol. 2014; 15(12). doi: 10.1186/s13059-014-0550-8.

34. Benjamini Y, Hochberg Y. Controlling the flase discovery rate-a practical and powerful approach to multiple testing. J Royal Stat Soc Series B-Methodol. 1995; 57(1):289-300.

35. Bohm G, Amarante L, Bohm E, Rombaldi C, Genovese M. Glyphosate Influence on the Soil Microorganism Sensibility, Physiological Parameters of the Plant, Isoflavones and Residues in the Seeds and Soil. J Agr Ecol Res Intl. 2016; 5(4):1-12. doi:10.9734/JAERI/2016/22186.

36. Xu BJ, Chang SKC. A comparative study on phenolic profiles and antioxidant activities of legumes as affected by extraction solvents. J Food Sci. 2007; 72(2):S159-S166. doi: 10.1111/j.1750-3841.2006.00260.x.

37. Jia Z, Tang MC, Wu JM. The determination of flavonoid contents in mulberry and their scavenging effects on superoxide radicals. Food Chem. 1999; 64(4):555-559. doi: 10.1016/s0308-8146(98)00102-2.

38. Giusti M, Wrolstad RE. Characterization and Measurement of Anthocyanins by UV-Visible Spectroscopy. In: Cur.Protocols Food Anal.Chem. doi: 10.1002/0471142913.faf0102s00 (2001)

39. Livak KJ, TSchmittgen D. Analysis of relative gene expression data using real-time quantitative PCR and the 2(T)(-Delta Delta C) method. Methods 2001; 25(4):402-408. doi: 10.1006/meth.2001.1262.

40. Gradillas A, Martínez-Alcázar MP, Gutierrez E, Ramos-Solano B, Garcia A. A rapid and novel strategy for the characterization of the complex triterpene saponin mixture present in the methanolic extract of blackberry leaves (Rubus cv. Loch Ness) by LC-MS-TOF. J Pharm Biomed Anal. 2019; 164:47–56 https://doi.org/10.1016/j.jpba.2018.10.014 .

41. Seema T, Mehta K, Singh D. Studies on the effect of plant growth promoting rhizobacteria (PGPR) on growth, physiological parameters, yield and fruit quality of strawberry cv. chandler. J Pharmacog Phytochem. 2018; 2 (7).

42. Kim JS, Lee J, Seo SG, Lee C, Woo SY, Kim SH. Gene expression profile affected by volatiles of new plant growth promoting rhizobacteria, Bacillus subtilis strain JS, in tobacco. Genes & Genomics 2015; 37 (4):387-397. doi: 10.1007/s13258-015-0267-4.

43. Samaniego-Gamez Y, Blancka RG, Tun-Suarez JM, Kantun-Can J, Reyes-Ramirez A, Cervantes-Diaz L. Bacillus spp. inoculation improves photosystem II efficiency and enhances photosynthesis in pepper plants. Chilean J. Agr. Res. 2016; 76(4). doi: 10.4067/s0718-2016.

44. Song GC, Ryu SY, Kim YS, Lee JY, Choi JS, Ryu CM. Elicitation of induced resistance against Pectobacterium carotovorum and Pseudomonas syringae by specific individual compounds derived from native Korean plant species. Molecules 2013; 18(10):12877-12895.

45. Gupta S, Garg V, Kant C, Bhatia S. Genome-wide survey and expression analysis of F-box genes in chickpea BMC Genomics. 2015; 16(1):67. doi: 10.1186/s12864-015-1293-y.

46. Pieterse CMJ, Zamioudis C, Berendsen RL,Weller DM, Van Wees SCM, Bakker PAHM. Induced Systemic Resistance by Beneficial Microbes Annu Rev Phytopathol. 2014; 52:347–75

47. Chepyshko H, Lai CP, Huang LM, Liu JH, Shaw JF. Multifunctionality and diversity of GDSL esterase/lipase gene family in rice (Oryza sativa L. japonica) genome: new insights from bioinformatics analysis. BMC Genomics. 2012;13:309. doi: 10.1186/1471-2164-13-309.

48. Gutierrez-Albanchez E, García-Villaraco A, Lucas JA, Gutierrez FJ, Ramos-Solano B. Priming fingerprint induced by Bacillus amyloliquefaciens QV15, a common pattern

in Arabidopsis thaliana and in field-grown blackberry. J Pl Interact. 2018; 13(1): 398-408, DOI: 10.1080/17429145.2018.1484187.

49. Ning Y, Liu W, Wang GL. Balancing Immunity and Yield in Crop Plants. Trends Plant Sci. 2017; doi:10.1016/j.tplants.2017.09.010.

50. Koskimaki JJ, Hokkanen J, Jaakola M, Suorsa M, Tolonen A, Mattila S, Pirttila AM, Hohtola, A. Flavonoid biosynthesis and degradation play a role in early defence responses of bilberry (Vaccinium myrtillus) against biotic stress. Eur J Plant Pathol. 2009; 125(4):629-640. doi: 10.1007/s10658-009-9511-6.

51. Makoi J, Belane AK, Chimphango SBM, Dakora FD. Seed flavonoids and anthocyanins as markers of enhanced plant defence in nodulated cowpea (Vigna unguiculata L. Walp.). Field Crop Res. 2010; 118(1):21-27. doi: 10.1016/j.fcr.2010.03.012.

52. García-Seco D, Zhang Y, Gutierrez-Mañero FJ, Martin C, Ramos-Solano B. Application of Pseudomonas fluorescens to blackberry under field conditions improves fruit quality by modifying flavonoid metabolism. PLoS ONE 2015; 10:23. doi: 10.1371/journal.pone.0142639.

53. Gutierrez- Albanchez E, Kirakosyan A, Bolling SF, García- Villaraco A, Gutierrez- Mañero FJ, Ramos- Solano B. Biotic elicitation as a tool to improve berry (Strawberry and Raspberry) extract potential on metabolic syndrome related enzymes in vitro. J Sci Food Agr. 2019; 99(6):2939-2946. doi.org/10.1002/jsfa.9507.

54. Taye-Desta K, Shin SC, Shim JH, Kim GS, Shin HC, Abd El-Aty AM. Flavonoid Variations in Pathogen-Infected Plants. Front Nat Prod Chem. 2016; 2:3-49.

55. Ahuja I, Kissen R, Bones AM. Phytoalexins in defense against pathogens. Trends Plant Sci. 2012; 17 (2):73-90. doi: 10.1016/j.tplants.2011.11.002.

56. Mierziak J, Kostyn K, Kulma A. Flavonoids as important molecules of plant interactions with the environment. Molecules 2014; 19(10):16240 -16265.

57. Weidenbörner M, Jha HC. Antifungal activity of flavonoids in relation to degree of hydroxylation, methoxylation and glycosidation. Acta Hortic. 1993; 381:702-709 DOI: 10.17660/ActaHortic.1994.381.102.

58. Cushnie T, Tim P, Lamb AJ. Antimicrobial activity of flavonoids. Intl. J. Antimicrob Ag. 2005; 26(5):343-356.

59. Parvez MM, Tomita-Yokotani K, Fujii Y, Konishi T, Iwashina T. Effects of quercetin and its seven derivatives on the growth of Arabidopsis thaliana and Neurospora crassa. Biochem System Ecol. 2004; 32(7):631-635. doi: 10.1016/j.bse.2003.12.002.

60. Nutzmann HW, Huang A, Osbourn A. Plant metabolic clusters - from genetics to genomics. New Phytol. 2016; 211(3):771-789. doi: 10.1111/nph.13981.

61. Liu J, Osbourn A, Pengda MA. MYB Transcription Factors as Regulators of Phenylpropanoid Metabolism in Plants. Mol. Plant 2015; 8(5):689-708. doi: 10.1016/j.molp.2015.03.012.

62. Shibu Prasanth SCR, Chandran P. Phytochemical and antimicrobial analysis of leaf samples of different Rubus species. Intl J Chem Tech Res. 2017; 10(4):359-368.

Supporting information (IF APPLICABLE)

S1 Fig. Blackberry fruits chromatograms. Overlaid Chromatograms (positive and negative ion mode) obtained from LC/MS/TOFF analysis of the methanolic extract of BlackBerry fruit samples. Control samples are represented in green while QV15 samples appear in red

S1 Table. Number of mappable samples and paired readings per sample

S2 Table. Expression of Glutathione S transferases. Glutathione S transferases gene expression analyzed by RT-qPCR in leaves and fruit. Asterisks indicate significant differences, according to Fisher test ($p<0.05$).

S3 Table. Primers designed to RT-qPCR expression analysis

S1 File. Differentially expressed genes in leaves

S2 File. Differentially expressed genes in fruits

Figure Legends

Figure 1. Biosynthesis of anthocyanins, flavonols and catechins via the flavonoid pathway in *Rubus* cv. Loch Ness. Phenylalanine ammonio-lyase (RuPAL1 and RuPAL2), Cinammate-4-hydroxylase (RuC4H), 4-coumaryl-CoA ligase (Ru4CL), Chalcone synthase (RuCHS), Chalcone Isomerase1 (RuCHI1), Chalcone Isomerase2 (RuCHI2), Flavonol-3-hydroxylase (RuF3H), Flavonoid 3´5´hydroxylase (RuF3´5´H), Flavonoid 3´hydroxylase (RuF3’H), Flavonol synthase (RuFLS), Leucoanthocyanidin reductase (RuLAR), Anthocyanidin reductase (RuANR), Dehydroflavonol reductase

(RuDFR), Anthocyanidin synthase (RuANS), Flavonol and Anthocyanidin Glycosiltransferases (RuFLS and RuAGT).

Figure 2. Heat Map and unsupervised hierarchical clustering by sample; top 50 genes with the largest coefficient of variation based on FPKM counts, a) in leaves b) in fruits

Figure 3 Venn diagrams of overexpressed and common genes in blackberry leaves (a) and fruits (b) from control and QV15-treated plants

Figure 4. Overlaid Chromatograms (positive and negative ion mode) obtained from LC/MS/TOFF analysis of the methanolic extract of Blackberry leaf samples. Control samples are represented in Green while QV15 samples appear in red

Figure 5. Flavonol-Anthocyanin Pathway gene expression analyzed by RT-qPCR in blackberry leaves. Phenylalanine ammonio-lyase (*RuPAL1* and *RuPAL2*), Cinammate 4 hydroxylase (*RuC4H*), 4-coumaryl-CoA ligase (*Ru4CL*), Chalcone synthase (*RuCHS*), Chalcone Isomerase1 (*RuCHI1*), Chalcone Isomerase2 (*RuCHI2*), Flavonol-3-hydroxylase (*RuF3H*), Flavonoid 3´5´hydroxylase (*RuF3´5´H*), Flavonoid 3´hydroxylase (*RuF3'H*), Flavonol synthase (*RuFLS*), Leucoanthocyanidin reductase (*RuLAR*), Anthocyanidin reductase (*RuANR*), Dehydroflavonol reductase (*RuDFR*), Anthocyanidin synthase (*RuANS*). Insert: Flavonol-anthocyanin pathway regulatory genes. Asterisks indicate significant differences, according to Fisher test ($p<0.05$).

Figure 6. Flavonol-Anthocyanin Pathway gene expression analyzed by RT-qPCR in blackberry fruits. Phenylalanine ammonio-lyase (*RuPAL1* and *RuPAL2*), Cinammate 4 hydroxylase (*RuC4H*), 4-coumaryl-CoA ligase (*Ru4CL*), Chalcone synthase (*RuCHS*), Chalcone Isomerase1 (*RuCHI1*), Chalcone Isomerase2 (*RuCHI2*), Flavonol-3-

hydroxylase (*RuF3H*), Flavonoid 3´5´hydroxylase (*RuF3´5´H*), Flavonoid 3´hydroxylase (*RuF3’H*), Flavonol synthase (*RuFLS*), Leucoanthocyanidin reductase (*RuLAR*), Anthocyanidin reductase (*RuANR*), Dehydroflavonol reductase (*RuDFR*), Anthocyanidin synthase (*RuANS*). Insert: Flavonol-anthocyanin pathway regulatory genes. Asterisks indicate significant differences, according to Fisher test ($p<0.05$).

Figure 1. Pathway
Click here to download Figure Figure 1.tif

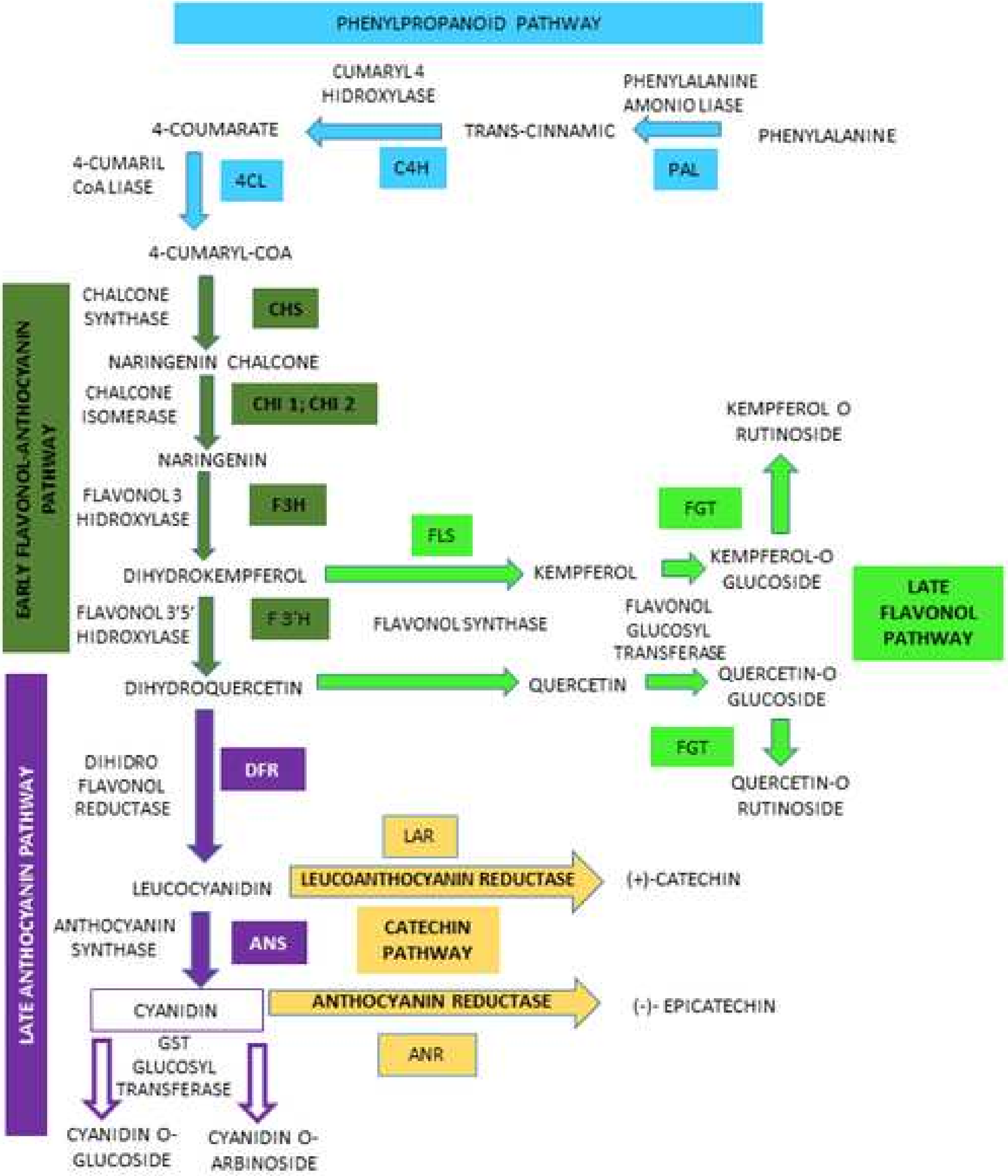

Figure 2. Heatmaps

Click here to download Figure Figure 2 heatmaps.tif

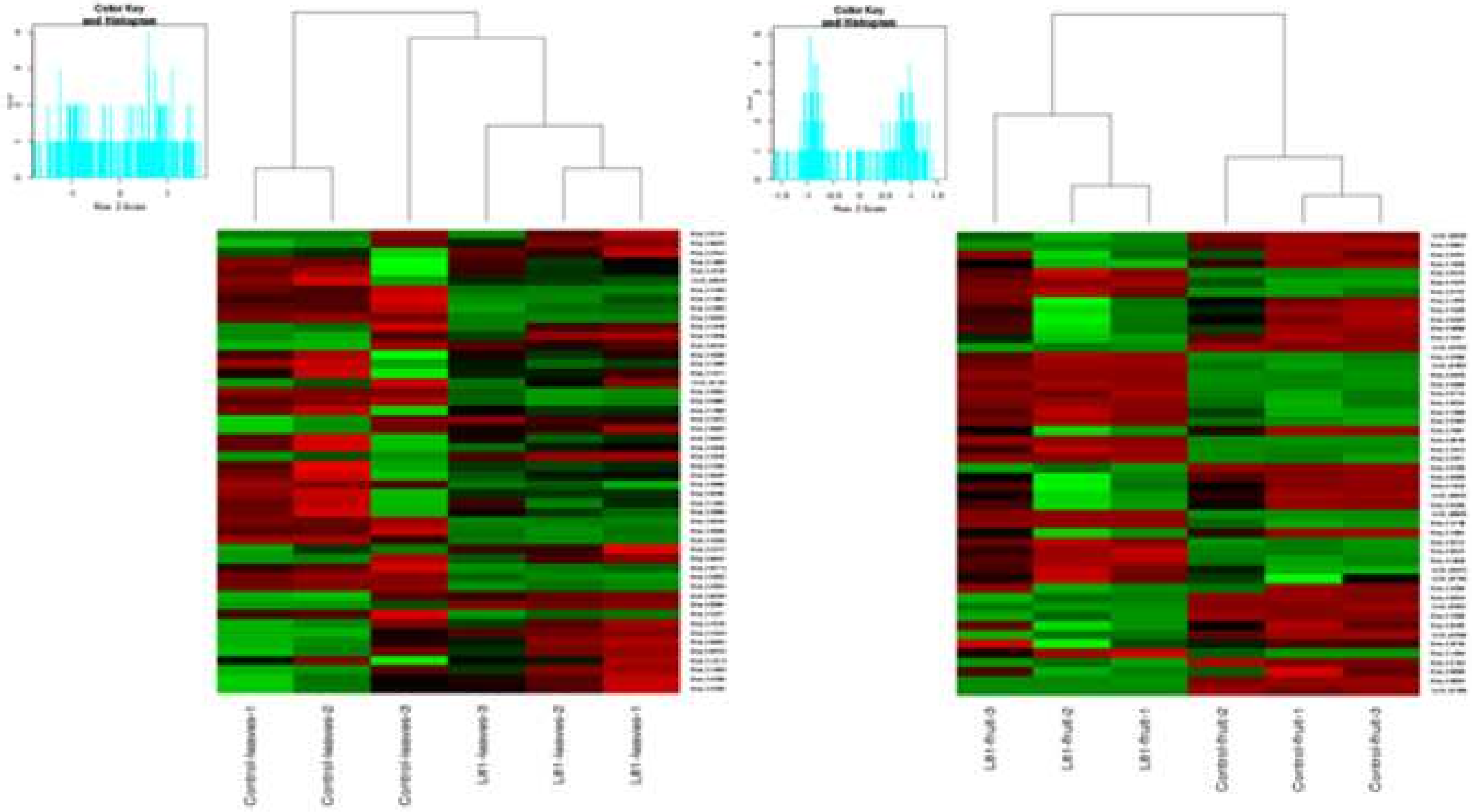

Figure 3. Venn diagrams

Click here to download Figure Figure 3 ven diagrams.tif

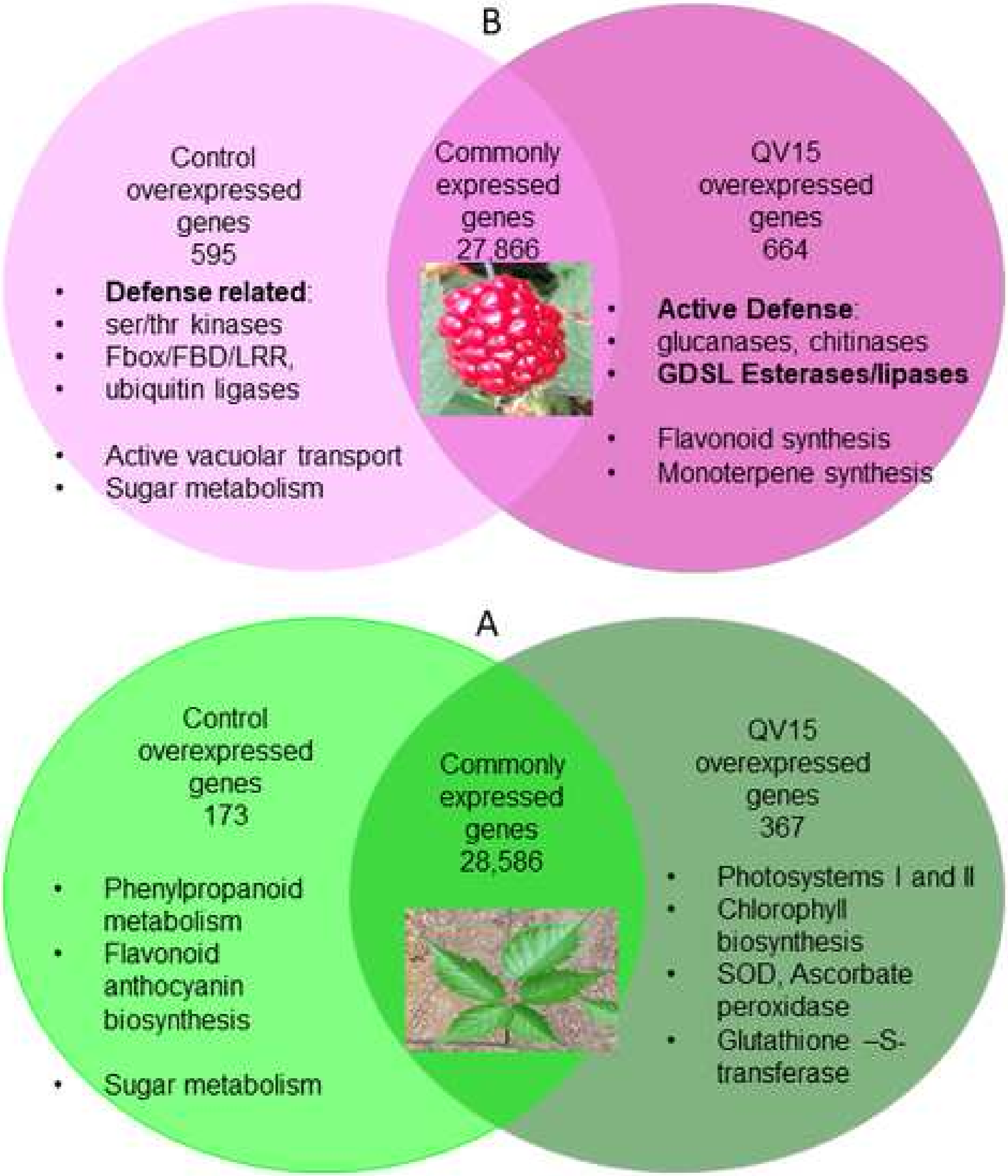

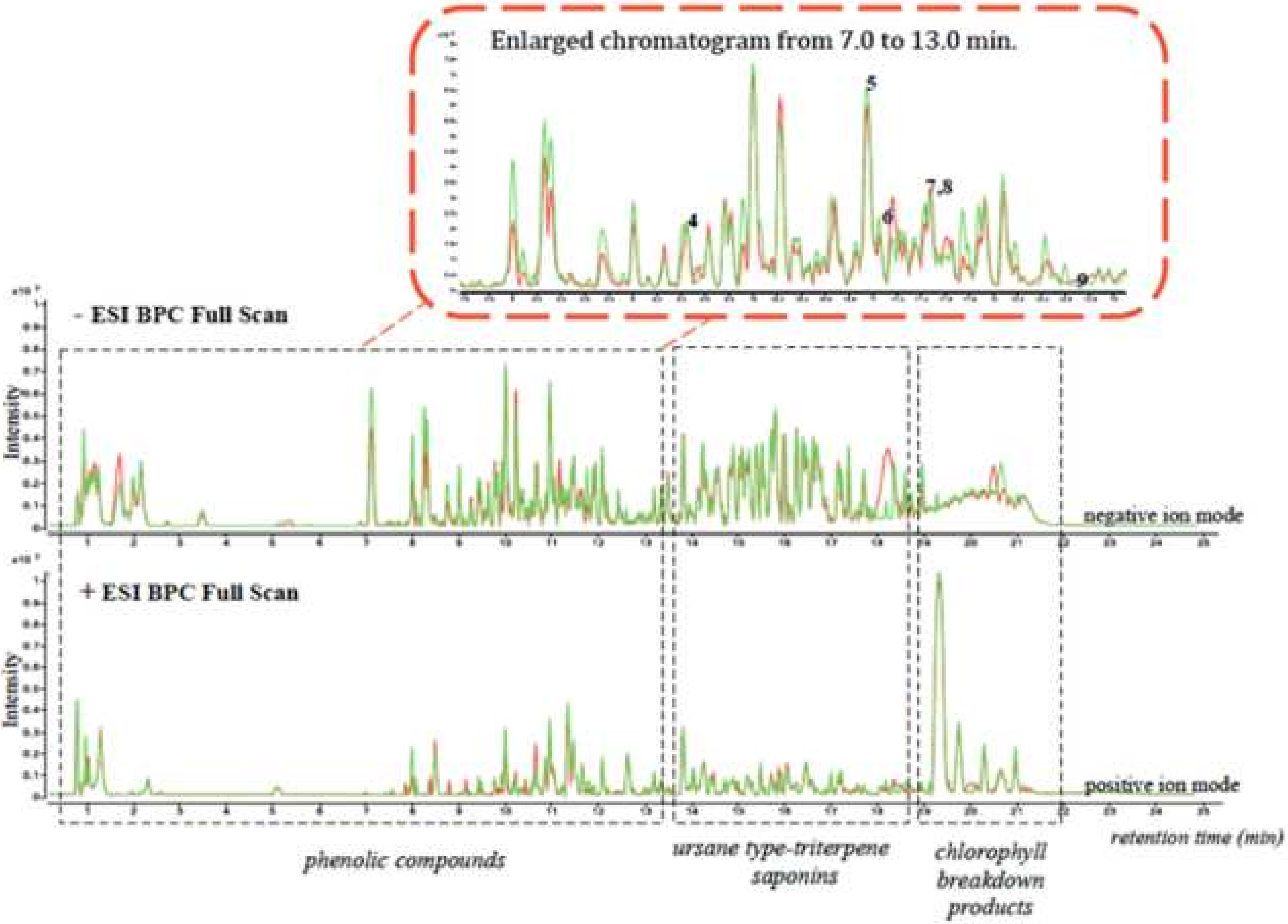

Enlarged chromatogram from 7.0 to 13.0 min.
5
4
6
7,8
9
- ESI BPC Full Scan
Intensity
negative ion mode
+ ESI BPC Full Scan
Intensity
positive ion mode
retention time (min)
phenolic compounds
ursane type-triterpene saponins
chlorophyll breakdown products

 Click here to download Figure Figure 5 Differential Gene expression leaves.tif

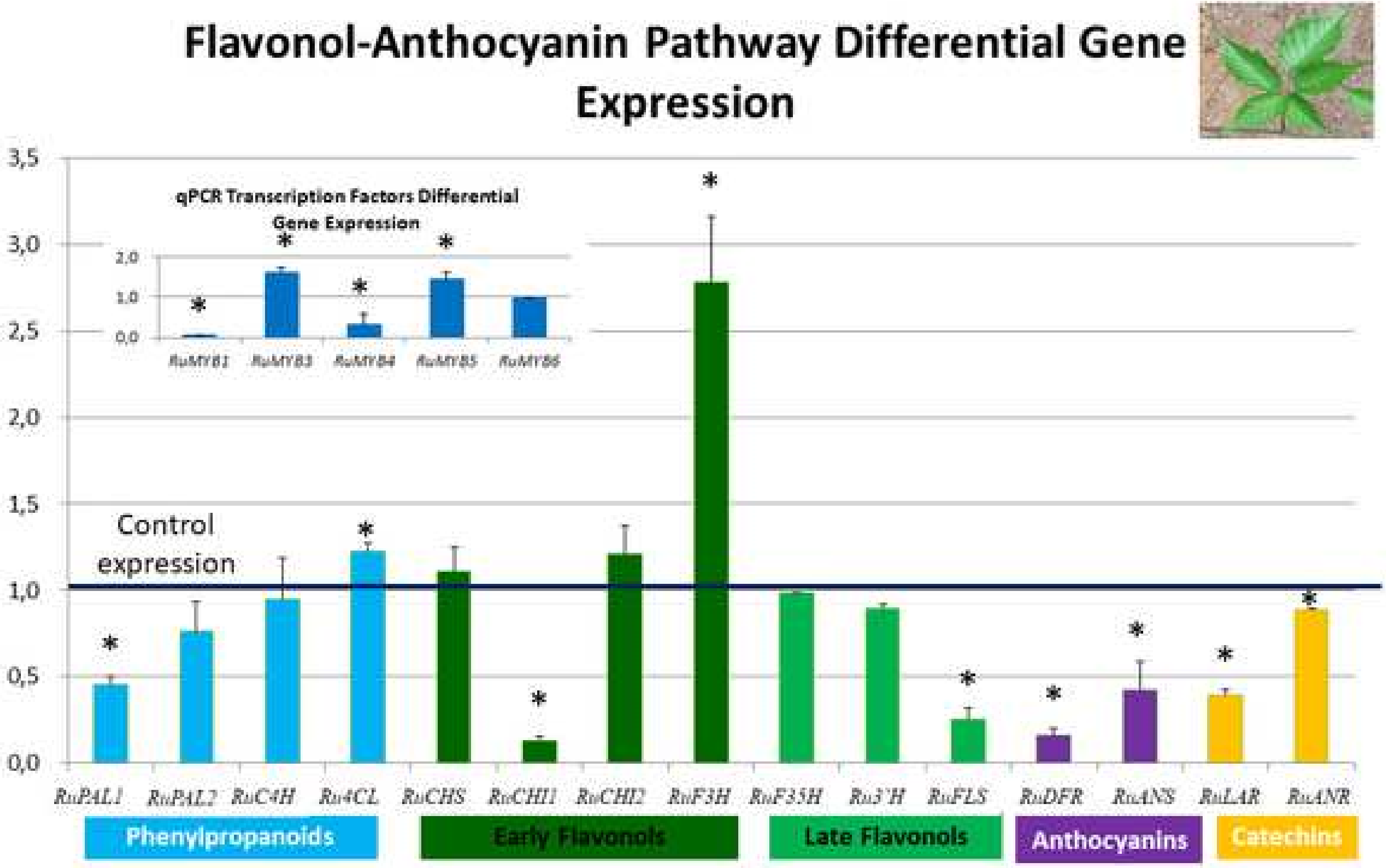

Figure 6. Gene expression fruits

Click here to download Figure Figure 6 Differential gene expression fruits.tif

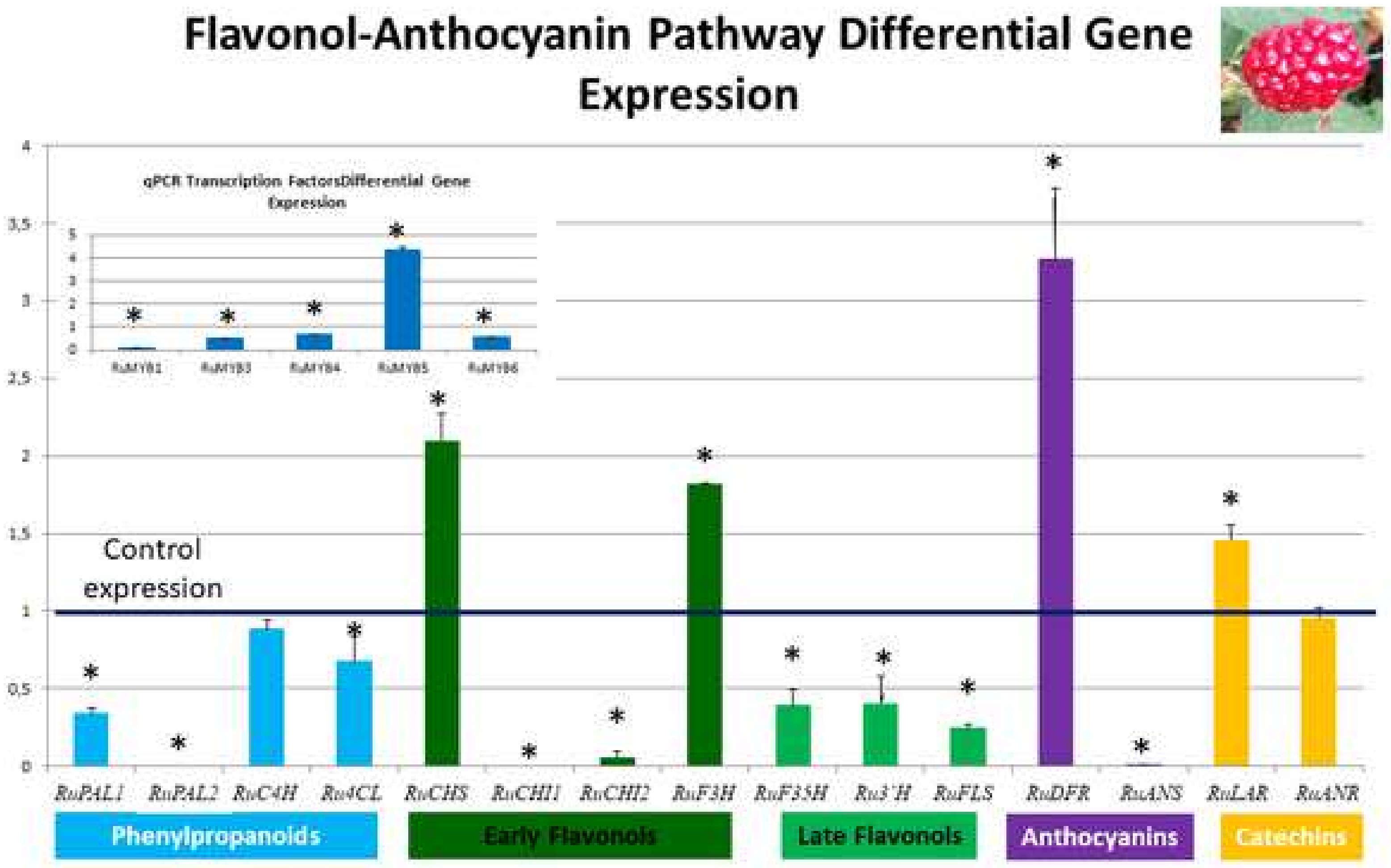

S1 figure. chromatogram fruits

Click here to access/download
Supporting Information
1S Figure fruit chromatograms.tif

S1 Table

Click here to access/download
Supporting Information
S1_Table. Ramos-Solano.docx

Click here to access/download
Supporting Information
S2_Table. Ramos-Solano.docx

S3 Table

Click here to access/download
Supporting Information
S3_Table. Ramos-Solano.docx

S1 File gene expression leaves

Click here to access/download

Supporting Information

S1_File Leaves differentially expressed genes.xlsx

S2 File gene expression fruits

Click here to access/download
Supporting Information
S2_File Fruits Differentially expressed Genes.xlsx
